# Distinct aging-related profiles of allocentric knowledge recall following navigation in an immersive, naturalistic, city-like environment

**DOI:** 10.3389/fnagi.2026.1746016

**Published:** 2026-06-02

**Authors:** Yasmine Bassil, Anisha Kanukolanu, Emma Funderburg, Emily Z. Cui, Thackery I. Brown, Michael R. Borich

**Affiliations:** 1 Neuroscience Graduate Program, Graduate Division of Biological and Biomedical Sciences, Emory University, Atlanta, GA, United States; 2 Division of Physical Therapy, Department of Rehabilitation Medicine, Emory University School of Medicine, Atlanta, GA, United States; 3 College of Sciences, Georgia Institute of Technology, Atlanta, GA, United States; 4 School of Psychology, Georgia Institute of Technology, Atlanta, GA, United States; 5 Center for Research and Education in Navigation, Georgia Institute of Technology, Atlanta, GA, United States

**Keywords:** aging, allocentric, cognitive map, spatial navigation, virtual reality, navigation

## Abstract

Aging-related declines in spatial navigation pose significant challenges for older adults’ independence and quality of life. Among navigational deficits, older adults have been shown to demonstrate deficits in utilizing allocentric (i.e., world-centered) information and rely on egocentric (i.e., body-centered) cues during navigation, resulting in reference frame bias. We investigated naturalistic navigation performance and allocentric knowledge formation in younger adults (*N =* 30) and older adults (*N =* 30) using a city-like virtual reality wayfinding task (*NavCity*) across multiple within-session exposures, paired with a *NavCity* Allocentric Representation Assessment (NARA). Older adults demonstrated significantly lower navigation performance compared to younger adults including traveling greater distances, taking longer navigation times, moving at slower speeds, and exhibiting longer dwell times while navigating. Despite aging-related differences, both age groups showed similar rates of performance improvement across exposure blocks. Following repeated *NavCity* exposures, older adults demonstrated lower allocentric knowledge formation, but both age groups demonstrated significant associations with navigation performance. Notably, substantial heterogeneity was observed within the older adult group, with a bimodal distribution in NARA scores that split older adults into higher- and lower-performing subgroups, which corresponded to differences in navigation performance independent of chronological age. Higher-performing older adults exhibited navigation performance and allocentric knowledge formation comparable to younger adults, while lower-performing older adults showed persistent deficits in both navigation performance and allocentric knowledge formation despite repeated exposures. These findings suggest that aging-related navigation decline is not uniform and highlight the possibility of combined virtual navigation and allocentric assessment tasks as potential sensitive, early indicators of aging-related declines in spatial navigation ability.

## 1 Introduction

Aging is a universal human experience that inevitably changes how we think, move, and navigate through the world around us. With advancing age, individuals experience gradual declines in multiple cognitive domains, including processing speed, working memory, and executive function (Park and Reuter-Lorenz, 2009; Salthouse, 2019). Within this broader pattern of cognitive aging, *spatial navigation ability* – our ability to use information from the environment to find our way from one place to another – declines substantially (Klencklen et al., 2012; Lester et al., 2017; Lithfous et al., 2013; Moffat, 2009; Moffat and Resnick, 2002), with effect sizes often exceeding those seen in other cognitive domains (Techentin et al., 2014). Older adults often report and exhibit difficulties finding their way in unfamiliar environments (Burns, 1999; Heward et al., 2023; Marquez et al., 2017; Xu et al., 2024), with navigation difficulties contributing to reduced independence and mobility, anxiety about exploring new places, and social isolation (Chee, 2023; Muffato et al., 2022, 2023; Phillips et al., 2013; van der Ham et al., 2013). Importantly, spatial navigation impairments and progressive topographical disorientation are among the earliest detectable signs of aging-related cognitive decline and neurodegenerative pathologies, often emerging prior to and predicting clinical diagnosis of mild cognitive impairment or Alzheimer’s disease (Cerman et al., 2018; Gazova et al., 2012; Klein et al., 1999; Levine et al., 2020; Verghese et al., 2017), and may be used as a behavioral biomarker across stages of aging (Laczó et al., 2009; Plácido et al., 2022; Serino et al., 2014; Tangen et al., 2015).

The specificity of aging-related navigational deficits that extend beyond general cognitive slowing has now been extensively characterized, including fundamental changes in how older adults encode and utilize spatial information. While older adults may maintain aspects of spatial perception and visuospatial processing (Lester et al., 2017; Norman et al., 2009, 2015), they demonstrate marked deficits in more complex aspects of navigation, including wayfinding in unfamiliar environments (Head and Isom, 2010; Kirasic, 1991; Xu et al., 2024), forming and updating mental representations of environments (Iaria et al., 2009; Moffat et al., 2006; Moffat and Resnick, 2002), and flexibly switching between navigation strategies (Harris et al., 2012; Harris and Wolbers, 2014; Rodgers et al., 2012).

However, a particularly important aspect of this decline involves spatial reference frames used during navigation. Successful navigation typically relies on two complementary reference frames: egocentric reference frames, which encode viewer-dependent, body-centered spatial relationships, and allocentric reference frames, which encode viewer-independent, world-centered relationships between environmental landmarks (Burgess, 2006; Colombo et al., 2017; Klatzky, 1998; Moffat et al., 2006). These reference frames are supported by concrete spatial cues from the environment, which may also be described as egoformative (i.e., body-relative) and alloformative (i.e., world-relative) cues, which then lend to the formation of egocentric and allocentric reference frames, respectively (Starrett et al., 2023). Importantly, these reference frames should not be viewed as a strict dichotomy, but rather as endpoints on a spectrum of spatial representations (Ekstrom et al., 2017; Starrett and Ekstrom, 2018; Wang, 2017). While debate continues about how these representations are encoded and stored in memory (Ladyka-Wojcik and Barense, 2021), effective navigation is shown to require the integration of spatial information across the egocentric-to-allocentric reference frame continuum, flexibly using information based on environmental demands (Ekstrom et al., 2014; Gramann et al., 2010; Lester et al., 2017; Mou et al., 2006). In the context of aging, older adults show a demonstrable bias toward utilizing egocentric reference frames, with robust deficits in using allocentric information (Colombo et al., 2017; Gazova et al., 2012; Laczó et al., 2018; Rodgers et al., 2012). This reduced flexibility in reference frame use in older adults, termed “reference frame bias,” represents a key navigational deficit in aging. Such bias significantly contributes to decreased navigational performance (Lester et al., 2017) and may serve as a quantifiable behavioral marker for identifying individuals at risk of future cognitive decline (Coughlan et al., 2018; Laczó et al., 2017).

Central to these impairments in allocentric spatial processing is the formation of cognitive maps—internal, map-like, allocentric representations of environmental layout that encode spatial relationships between landmarks independent of one’s viewpoint (Ekstrom and Isham, 2017; O’Keefe and Nadel, 1978; Tolman, 1948). These cognitive maps, also referred to as survey knowledge, represent an important aspect of spatial knowledge that develops through navigation experience and enables flexible wayfinding behaviors such as taking shortcuts, navigating from novel starting points, and inferring spatial relationships between landmark pairings (Montello, 1998; Siegel and White, 1975). Cognitive map formation is classically linked to the hippocampus and a broader network of distributed brain regions that support allocentric spatial representation (Epstein and Baker, 2019; O’Keefe and Nadel, 1978). Studies measuring cognitive map formation have used tasks assessing survey knowledge (i.e., map drawing, or sketch mapping) and direction estimation, which demonstrate that older adults form less accurate survey representations of navigated environments compared to younger adults (Head and Isom, 2010; Moffat and Resnick, 2002; Zhong and Moffat, 2016), even when route-based performance may be relatively preserved (Cushman et al., 2008). This dissociation suggests that aging selectively impairs the transformation of first-hand navigation experiences into integrated, map-like, allocentric spatial representations. The inability to form robust mental representations of space as cognitive maps has important implications for navigation ability, as it limits the ability to plan efficient routes, recognize environmental relationships, and adapt flexibly to changes in the environment.

Aging-related deficits in cognitive map formation and allocentric spatial processing are well-documented (Harris et al., 2012; Head and Isom, 2010; Iaria et al., 2009; Moffat, 2009; Moffat et al., 2006; Moffat and Resnick, 2002), with prior work testing diverse methodological approaches and cognitive processes (Simonet et al., 2025). Studies have also examined performance across multiple temporal scales, following single exposures or limited trials in novel environments (Chrastil and Warren, 2013; Moffat and Resnick, 2002; Weisberg et al., 2014), within-session learning across repeated trials (Kober et al., 2013; Mitolo et al., 2017; Nemmi et al., 2017; Wiener et al., 2013), and long-term consolidation of real-world spatial memories over months or years (Ishikawa and Montello, 2006; Lövdén et al., 2012; Woost et al., 2018). However, existing evidence on spatial skill acquisition and spatial learning trajectories presents a mixed picture.

Many studies demonstrate that younger adults can improve navigation performance with practice and develop survey knowledge of navigated environments (Allison and Head, 2017; Bassil et al., 2025; Gazova et al., 2013; Weisberg et al., 2014; Zhong et al., 2017), as one may expect. Despite general aging-related navigation deficits, older adults also show improvements in at least one spatial ability-related outcome following training interventions (Fricke et al., 2022; Gazova et al., 2013). However, regardless of age, other work reveals persistent deficits in allocentric perspective-taking tasks despite extensive repeated exposure—ranging from months of learning in real-world environments (Ishikawa and Montello, 2006; Moeser, 1988; Thorndyke and Hayes-Roth, 1982) to multiple training sessions in virtual environments (Münzer et al., 2006; Ruddle et al., 1997). Critically, improvements in route-based performance do not necessarily translate into enhanced survey knowledge (Taylor et al., 1999; Zhang et al., 2012), suggesting a dissociation between different types of spatial learning, which may be important to characterize with repeated exposure or training.

When it comes to trajectories of performance improvement and spatial learning, the degree to which healthy older adults may demonstrate improvement rates comparable to younger adults remains an active area of investigation. Some studies studying this across age groups demonstrate that both younger and older adults improve at similar rates in novel environments with repeated trials (Gazova et al., 2013; Head and Isom, 2010; Lövdén et al., 2012; Moffat et al., 2001; Nemmi et al., 2017), a pattern that fundamentally distinguishes healthy cognitive aging from pathological conditions such as early-stage Alzheimer’s disease, where within-session performance improvement on spatial tasks is markedly impaired (Gazova et al., 2012; Hort et al., 2007; Laczó et al., 2009, 2011). While other studies have shown different rates of improvement between age groups (Iaria et al., 2009; Yamamoto and Degirolamo, 2012), this has been potentially attributed to the type of spatial information being acquired. However, though improvement rates in navigation performance may be comparable, age groups often fail to fully converge in absolute performance levels even with extensive training, particularly in passive navigation paradigms, non-immersive desktop-based environments, or traditional measures of spatial cognition (Baltes and Kliegl, 1992; Head and Isom, 2010; Lövdén et al., 2012; Moffat and Resnick, 2002; Nemmi et al., 2017). While active navigation produces larger memory enhancements in older than younger adults compared to passive navigation (Meade et al., 2019), and immersive virtual reality attenuates aging-related navigation differences compared to non-immersive desktop environments (Hill et al., 2024), whether combining these features can facilitate convergence between age groups with repeated training remains unknown.

Substantial individual differences further complicate this picture. Some individuals develop accurate configurational knowledge after just one or two exposures, while others show minimal improvement even after 10 or more learning trials (Ishikawa and Montello, 2006). Distinct profiles of navigation ability have been characterized in younger adults, specifically in their ability to form cognitive maps (Weisberg and Newcombe, 2016, 2018; Weisberg et al., 2014), which has also been correlated to cognitive measures such as visuospatial working memory capacity (Blacker et al., 2017) and self-reported sense of direction (Hegarty, 2002), but not general intelligence (Weisberg and Newcombe, 2016). Critically, practice alone does not guarantee improvement—younger adults with poor self-reported sense of direction have shown limited training effects on cognitive map formation with unsupervised practice, compared to those with average sense of direction (Ishikawa and Zhou, 2020). These inconsistencies may reflect variations in environmental complexity, mode of presentation (real-world versus virtual), the number and spacing of learning trials, and specific spatial abilities assessed, even in younger adults.

Additionally, substantial heterogeneity within older adult populations in cognition function has been well documented (Hultsch et al., 2002, 2011; Morse, 1993). Performance variability in spatial working memory is particularly high within older adult samples, with some individuals able to attain performance levels within the range of younger adults without showing signs of compensatory brain activation (Nagel et al., 2009). A study examining navigation strategy use found that aging-related differences were evident only when comparing younger adults to lower-performing older adults, while higher-performing older adults demonstrated spatial abilities comparable to their younger counterparts (Zhong et al., 2017). Individual visuospatial factors such as visuospatial working memory and sense of direction also contribute significantly to navigation performance variability (Meneghetti et al., 2022). Recent work suggests that older adults’ individual differences in spatial ability arise from dissociable sources: some variation is linked to aging-related declines in neural mechanisms supporting spatial memory, while other variation reflects the fidelity of builtspatial representations that track individual performance regardless of age (Zheng et al., 2023). Understanding what distinguishes older adults who maintain spatial abilities from those who show decline has become increasingly important for identifying protective factors, developing targeted interventions, and detecting early markers of pathological aging. Spatial navigation deficits, particularly in allocentric processing, can precede clinical diagnosis of mild cognitive impairment and Alzheimer’s disease, making sensitive assessments of cognitive mapping ability potentially valuable for early detection.

Much existing navigation research employs desktop-based virtual environments or simplified spatial layouts that may lack ecological validity (Bishop and Rohrmann, 2003; Cushman et al., 2008; de Kort et al., 2003). While these controlled paradigms have isolated specific cognitive processes, immersive virtual reality environments have been shown to capture everyday navigation complexity and show close associations to real-world navigation (Campbell et al., 2009; Kourtesis and MacPherson, 2021; Parsons, 2015; Rizzo et al., 2004), as well as attenuate aging-related deficits (Hill et al., 2024). Furthermore, while map drawing tasks are commonly used to assess allocentric spatial knowledge, some assessments provide map learning from a birds-eye view perspective for space acquisition, rather than immersive first-person navigation (Thorndyke and Hayes-Roth, 1982), which is important as visualization method significantly impacts quality of spatial memory (Ye et al., 2023). Therefore, combining naturalistic, first-person navigational experiences with allocentric spatial assessment across repeated exposures remains largely unexplored in aging populations.

Repeated exposures are important for discovering environmental relationships and constructing integrated survey representations (Hilton and Wiener, 2023; Ishikawa and Montello, 2006; Montello, 1998; Siegel and White, 1975), yet few studies have combined naturalistic, immersive navigation with objective map-based assessments to examine how older adults build and retrieve allocentric spatial knowledge through within-session training. Significantly, this paradigm design enables characterization of allocentric knowledge formation in older adults. By doing so, it can be determined whether aging effects manifest as monotonic slowing of information acquisition throughout learning, initially-delayed learning that rapidly catches up once environmental schemas are formed and subsequent learning becomes facilitated, or plateau effects that suggest fundamental capacity limits. Within-session assessments also offer clinically feasible timeframes for evaluation and intervention for aging-related navigation deficits. Therefore, such an approach is suited to understanding how aging affects the dynamic interplay between self-directed navigation, route learning, and the emergence of survey knowledge under conditions that more closely approximate real-world navigation and show feasibility for potential future clinical administration.

The present study aimed to address gaps by examining within-session improvement in spatial navigation performance across three repeated navigation exposures in younger and older adults using *NavCity*, an immersive, naturalistic, city-like virtual environment. We assessed multiple dimensions of navigation behavior and their relationship to topographical, allocentric knowledge formation, or cognitive map formation, assessed via a map-based recall task to measure aging effects on the transformation of spatial information from an immersive, first-person navigational experience. We hypothesized that older adults would demonstrate overall lower navigation performance, compared to younger adults, across repeated exposures to a novel virtual environment, but that improvement in performance across exposures would be similar between age groups. Lastly, we hypothesized that individuals with better overall navigation performance would demonstrate greater allocentric spatial knowledge of the environment following exposures regardless of age, supporting a theoretical framework in which configural memory acquisition is a process slowed, but not bounded, by aging, suggesting that individual differences remain the dominant influential factor for allocentric information utilization. Additionally, based on prior work showing that some older adults exhibit preserved cognitive performance comparable to younger adults despite advancing age, along with our current work showing significant aging-related variability in performance, we further tested for evidence of individual differences in older adult performance that may differentiate older adults into distinct profiles of cognitive aging. Such profiles in spatial memory acquisition could be used to target different cognitive subgroups in older adults who may be on different cognitive aging trajectories with significant implications for quality of life and independence and warrant further research.

## 2 Materials and methods

### 2.1 Participants

A total of 30 neurotypical young adults (YAs) (ages: 18–35, mean (SD) = 24.34 (2.88); W = 17, M = 13) and 30 neurotypical older adults (OAs) (ages: 60+, mean (SD) = 69.01 (5.66); W = 19, M = 11) were recruited from participant databases and surrounding community locations in Atlanta, Georgia and retained in the final study sample.

Initial eligibility screening included: (1) no history of neurological disorders, major neurological events (e.g., stroke, seizures, traumatic brain injury), musculoskeletal impairments, or chronic conditions (e.g., autoimmune conditions, chronic fatigue, diabetes); (2) no current chronic pain diagnosis; (3) no recent head trauma (i.e., mild concussion within 3 months); (4) no major uncorrected visual impairments (e.g., glaucoma, cataracts); (5) ability to read instructions clearly in virtual reality (VR); (6) minimum 8th grade education; (7) fluent English proficiency; and (8) age within target ranges (18–35 for YAs; 60 + for OAs).

After being recruited, participants were excluded from the final dataset if they met either of the following exclusion criteria: (1) Mini-Cog score < 3, indicating possible cognitive impairment (Borson et al., 2000) (0 excluded), or (2) a change in pre- to post-VR Simulator Sickness Questionnaire (SSQ) score ≥ 10, indicating notable VR-induced symptoms (Bouchard et al., 2021; Kennedy et al., 1993) (0 excluded). The SSQ was administered before and after VR to monitor for sickness symptoms, with totals computed as the unweighted sum of all 16 items (theoretical range 0–48; Bouchard et al., 2021).

An additional 6 individuals were originally recruited (total of 66 participants) but were not included in the final sample, for the following reasons: 3 OAs revealed information that disqualified them from eligibility (i.e., musculoskeletal impairment, cane usage) upon completing the medical history questionnaire; 2 YA datasets were excluded due to technical difficulties (i.e., loss of recorded data); and 1 OA was unable to complete the *NavCity* virtual reality task.

Sample size determination was based on power analysis assuming large effect sizes (d = 0.8), typical of prior meta-analytic research in aging effects on spatial ability (Plácido et al., 2022; Techentin et al., 2014), yielding approximately 26 participants per group for 80% power (*α* = 0.05). We recruited 30 per group to account for potential attrition. The study protocol was approved under the Emory University Institutional Review Board. All study sessions are held in the Neural Plasticity Research Lab in the Emory Rehabilitation Hospital in Atlanta, Georgia.

### 2.2 Experimental procedure and design

#### 2.2.1 Demographics, questionnaires, and cognitive tasks

All procedures followed protocols established in prior work (Bassil et al., 2025). Participants attended a single two-hour experimental session in the Neural Plasticity Research Laboratory at Emory Rehabilitation Hospital at Emory University.

The session began with questionnaires and self-report measures, which included: a study-specific questionnaire for demographic information, lifestyle habits, and medical history collection, the Mini-Cog (Borson et al., 2000) for cognitive screening, the Pittsburgh Sleep Quality Index (PSQI) (Buysse et al., 1989) for sleep quality assessment, and the Santa Barbara Sense of Direction Scale (SBSOD) (Hegarty, 2002) for self-reported navigational ability. The Stanford Sleepiness Scale (SSS) (Hoddes et al., 1973) was administered at the very beginning and end of the session to assess changes in daytime sleepiness. The Simulator Sickness Questionnaire (SSQ) (Kennedy et al., 1993) was administered before and after VR exposure to monitor VR-induced symptoms.

Cognitive assessments were conducted using a KINARM Endpoint Lab (Kinarm Standard Tests™, BKIN Technologies) (Scott, 1999) to characterize aging-related differences in cognitive functions relevant to spatial navigation. These included the Trail Making Test A and B (Bowie and Harvey, 2006; Corrigan and Hinkeldey, 1987) to measure processing speed and cognitive set-shifting and the Corsi Blocks task (Berch et al., 1998) to assess visuospatial working memory.

#### 2.2.2 Virtual reality familiarization

**Figure 1:**
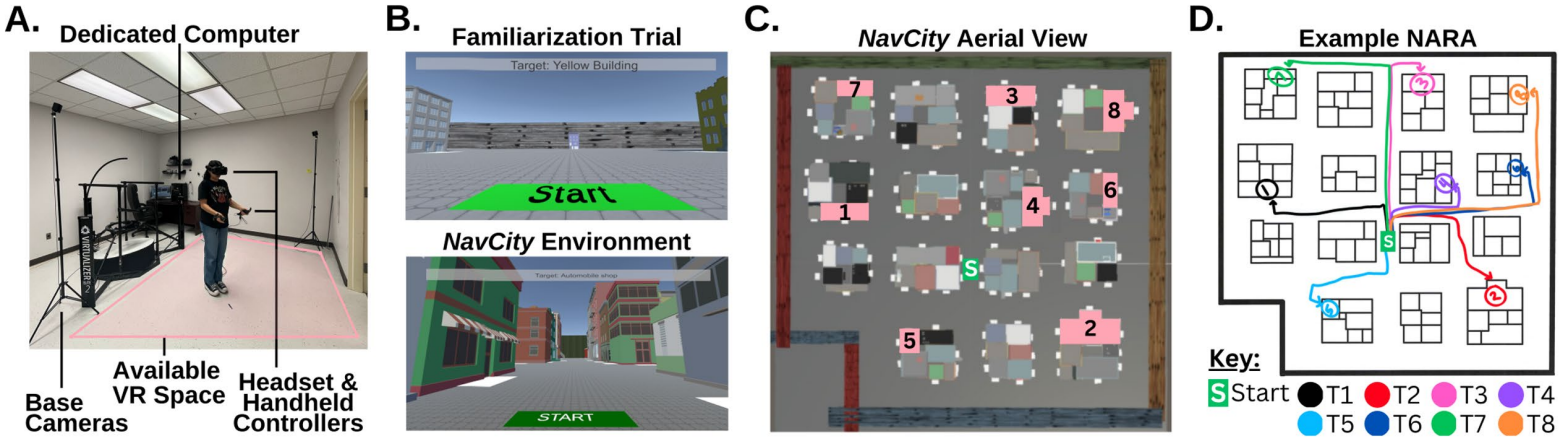
Experimental setup and virtual reality navigation environment. **(A)** Physical laboratory setup showing available lab space and equipment for the head-mounted display (HMD) VR system, including a dedicated computer system, handheld controllers, a head-mounted headset used for immersive navigation. **(B)** Ground-level perspective views of the VR familiarization trial (top) and *NavCity* virtual environment (bottom). **(C)** Aerial view of the *NavCity* VR environment showing the spatial layout of 8 target buildings distributed throughout the virtual city grid, as well as the marked ‘Start’ location. Building numbers (1–8) indicate the order of presentation within each block, with increasing intended difficulty – higher numbers reflect greater distance from Start and more complex routes (see Methods). **(D)** Example *NavCity* allocentric representation assessment (NARA), showing sample traces of paths and target building markings. NARA is a pen-and-paper assessment that participants complete after *NavCity* navigation to indicate memory of target building placement and path taken from the ‘Start’ after repeated *NavCity* exposure.

Following cognitive assessments, participants underwent a VR familiarization protocol using a head-mounted display (HMD) VR system (Valve Index VR Kit, Valve Corporation) (Figure 1A). The protocol included standardized instruction on headset use, controller use, and movement protocols within the VR environment, following previously established procedures (see Bassil et al., 2025) for full protocol details). Briefly, the familiarization trial included an open-space environment with similar visual aesthetics to the main navigation task, but only containing 3 simple, generic target buildings in plain sight (Figure 1B, top). During this trial, participants learned the teleportation-based locomotion system, where they used handheld controllers to move through the virtual environment via short, step-like teleportations. Specifically, participants pressed and held a button on the controller to project a visual teleportation arc into the environment, with the landing location indicated by a marker at the arc’s endpoint. Participants aimed the controller to adjust the direction and distance of the teleportation target, releasing the button to execute the teleport to the marked location. Teleportation distance was capped at a maximum of 10 VR units per teleport to maintain natural movement parameters. Participants also practiced reading target instructions displayed in their visual field and learned to indicate task completion by reaching designated white rectangles positioned in front of each building. The familiarization trial consisted of navigating to all three sample buildings, with participants allowed to repeat the trial as many times as needed to feel comfortable before proceeding to the main navigation task. All participants successfully completed the familiarization protocol.

#### 2.2.3 *NavCity* task

Following completion of the familiarization trial, participants completed the main navigation task for this study – the *NavCity* wayfinding task, followed by the corresponding *NavCity* Allocentric Representation Assessment (NARA), both previously established in Bassil et al. (2025). Participants completed 3 blocks of exposure to *NavCity*.

*NavCity* is a VR wayfinding task in a city-like environment designed to provide an immersive, real-world-like navigational experience similar to navigating through city blocks (Figure 1B, bottom). The environment was constructed using Unity (version 2020.3.16f1), with buildings arranged in a block-like layout with 8 unique target buildings placed throughout the city to serve as navigational destinations (Figure 1C). Target buildings contained unique, identifiable features, relevant signage, and visual cues to facilitate identification and were positioned to create routes with varying levels of difficulty across two features: Euclidean distance from the central Start location, and route complexity (e.g., number of turns required, placement near outer walls or corners requiring deeper traversal of the environment). Beyond these target buildings, the remaining cityscape included plain, non-specific buildings with similar design aesthetics to each other, as well as additional unique non-target buildings to replicate a realistic urban environment. To provide highly salient distal environmental cues for spatial orientation, the surrounding city walls were constructed with unique colors, similar to distal cues used in foundational navigation tasks, such as the Morris Water Maze (Morris, 1984), as well as other navigation tasks with virtual environments (Starrett et al., 2021; Vijayabaskaran and Cheng, 2022). One corner of the environment featured a distinctive inwards protruding corner to provide an additional landmark cue, comparable to a similar city-like VR environment (He et al., 2021) and other spatial tasks with corner cues (Jabbari et al., 2021; Newman and McNamara, 2022).

During each *NavCity* block, participants were instructed to navigate to each target building as quickly and safely as possible. Participants were given instructions at the top of their visual field to indicate the name of the target building for each trial. Participants started at a central ‘Start’ location, and after locating each building, participants were automatically returned back to the original start location. Each block consisted of all 8 target buildings presented in a fixed order designed to scale with difficulty features, with earlier targets located closer to the Start with simpler routes and later targets located farther from Start with more complex routes. We note that this ordering reflects the intended design of the environment rather than an empirically validated difficulty metric. Participants completed 3 exposure blocks (24 total trials) to assess spatial navigation performance (see Bassil et al., 2025) for full task protocol details).

Raw *NavCity* data outputted from the task included X-Z position in the environment and elapsed time, which were analyzed to calculate primary and secondary outcome measures. Primary outcome measures for *NavCity* performance included: (1) speed, calculated as total distance traveled divided by total navigation time (VR meters/s); (2) distance traveled, defined as the distance from the start block to the target building (in VR meters); and (3) navigation time, defined as the time elapsed between movement initiation and arrival at the target building (in seconds). All outcomes were calculated per target and averaged across targets to create navigational outcomes per block.

*NavCity* outcome measures may reflect different aspects of spatial navigation ability. Our primary outcomes include: (1) speed, which reflects the efficiency of movement through the virtual environment by combining both time-dependent and distance-dependent factors; (2) navigation time, a time-dependent measure that quantifies temporal efficiency in reaching target locations and may reflect individual differences in selective attention and perceptual processing during visual search (Ebaid and Crewther, 2019; Madden and Langley, 2003), as OAs often exhibit slower spatial information processing (Meng et al., 2019); and (3) distance traveled, a time-independent measure of spatial efficiency in reaching target locations. Shorter distances over repeated navigation blocks may also potentially reflect more efficient route selection and better memory formation (Daugherty et al., 2016; Gagnon et al., 2018; Moffat and Resnick, 2002).

Secondary outcome measures included: (1) dwell duration, defined as the average time spent stationary at each position; (2) teleportation count, defined as the number of teleportations used to navigate from start to target; and (3) teleport distance, defined as the average distance traveled per teleportation in VR meters. Our secondary outcome may also capture additional aspects of navigation behavior and VR adaptation. Dwell duration represents time-dependent pausing behavior that may reflect periods of spatial decision-making (Brunyé et al., 2018), similar to scanning or “pause-and-look” behavior in rodents (Monaco et al., 2014; Redish, 2016) or positional pausing and visual scanning in humans (Munion et al., 2019; Santos-Pata and Verschure, 2018), with longer dwell times potentially indicating more environmental scanning or reorientation at decision points, boundaries, or landmarks (Muessig et al., 2024), which may lead to more effective navigation (Ploran et al., 2014). Teleportation count and mean teleport distance are VR-specific, time-independent measures that may reflect individual differences in virtual locomotion strategies and comfort with the VR interface. Teleportation behavior may particularly distinguish between users who prefer frequent, short-distance movements versus those who use fewer, longer-distance teleportations to navigate the environment. Because teleportation count is inherently related to total distance traveled for a given path, it parly reflects the same spatial efficiency captured by distance traveled. However, when interpreted alongside mean teleport distance, it provides complementary information about the granularity of movement through the environment when using VR. These distinct measures allow for comprehensive assessment of how different aspects of spatial navigation and VR adaptation may vary between age groups.

#### 2.2.4 *NavCity* allocentric representation assessment (NARA)

Following completion of *NavCity*, participants then completed the *NavCity* Allocentric Representation Assessment (NARA) (Figure 1D), a pen-and-paper task designed to assess the ability to form topographical, allocentric spatial representations of the *NavCity* environment (see Bassil et al., 2025 for full protocol details). The NARA evaluates participants’ ability to transform first-person, viewer-dependent spatial information encoded during navigation into third-person, viewer-independent allocentric relationships between landmarks. Consistent with the view that egocentric and allocentric representations lie on a continuum rather than being strictly dichotomous, the NARA is designed to measure the output of this transformation – the accuracy of the viewer-indepdent, topologicl, map-like representation a participant produces – rather than inferring the specific use of strategy (e.g., imagined self-motion versus direct survey-level recall) used to generate it. As with other sketch-mapping and map-drawing tasks, scoring is based on the spatial fidelity of the drawn representation, not on the reference frame strategy used during retrieval. The resulting representation is often referred to as survey knowledge, a two-dimensional, map-like representation of an environment that provides an allocentric reference framework for spatial information (Wang et al., 2024; Warren, 2019).

Participants were seated at a nearby table and provided with an aerial, bird’s-eye view of the *NavCity* environment, with black outlines of buildings and walls and an “S” block to indicate the central location of the start position. For each of the 8 target buildings, participants used colored pens to mark the target building location on the aerial map and draw the path most representative of their route from the start location to that target building. All participants marked target buildings in the same order using the same color sequence, with no time restrictions imposed.

NARA scores were calculated primarily based on the accuracy of the marked building location, with the drawn path used in specific cases to adjust for partial credit (see below). Scoring followed established criteria (Bassil et al., 2025) using a 3-point scale per building: a score of 1 was awarded for correct target building identification; a score of 0.5 was given for partial credit when the incorrect building was marked but met one of the following qualitative spatial criteria: (1) the location of another target building, reflecting a target-identity switch error, (2) a building adjacent to the correct target, (3) a building directly opposite the correct target across a city block, (4) a mirrored global shape of the correct path and target location, (5) a rotated global shape of the correct path and target location, or (6) a building one block from the correct target, in cases where the drawn path overshot or undershot the correct target by one block, indicating incorrect path termination. A score of 0 was awarded when the marked building was incorrect and did not meet any partial credit criteria. Individual target building scores were summed to create a total NARA score (maximum possible score = 8).

### 2.3 Data analysis

All data cleanup, processing, and computation of outcome measures were conducted in Python (version 3.12.13), and all statistical analyses and figure generation were conducted in R using RStudio (version 2023.06.1). All analysis code is publicly available on GitHub: https://github.com/npresearchlab/navaging-paper. De-identified participant data are publicly available on the Open Science Framework: https://osf.io/qmwyk.

#### 2.3.1 Demographics, questionnaires, and cognitive tasks

Demographic characteristics collected from our study-specific questionnaire were compared between YA and OA groups. Self-reported demographic information included gender (all participants self-identified as either cisgender women or men), handedness (right-handed versus non-right-handed), VR experience (3-point scale: 0 = no prior use, 1 = minimal use or 1–3 lifetime exposures, 2 = recreational use or >3 lifetime exposures), video game usage (hours per week), and exercise frequency (hours per week). Self-reported lifestyle habits were also collected from the SBSOD and PSQI and calculated scores were also compared between YA and OA groups.

Performance on cognitive assessments was also compared between age groups, including total scores from the Corsi Block Test and completion time on the Trail Making Test Parts A and B. Difference between Trails A and B performance (B completion time - A completion time) was also calculated to isolate cognitive set-shifting ability from basic visuospatial processing speed. Pre- and post-session SSS scores were collected, as well as pre-and post-VR SSQ scores. SSQ total scores were calculated as the unweighted sum of all 16 item ratings (theoretical range: 0–48), following the approach recommended by Bouchard et al. (2021) for VR research. Change scores (post - pre) were also calculated for both SSS and SSQ measures.

Categorical variables (gender, handedness) were analyzed using chi-square tests or Fisher’s exact test when expected frequencies were below 5. Prior VR experience and SSS scores were compared using a Mann–Whitney U test, appropriate for single-item ordinal scales.

For all other measures, normality was assessed using Shapiro–Wilk tests and homogeneity of variance using Levene’s test. When distributional assumptions were met, independent samples *t*-tests were conducted; when violated, Mann–Whitney U tests were employed. Though the SBSOD, PSQI, and SSQ comprise individual items on ordinal scales, their summed total scores were treated as quasi-continuous variables. Validated multi-item Likert-type scales approximate interval-level measurement and can be appropriately analyzed with parametric tests when distributional assumptions are satisfied (Norman, 2010; Sullivan and Artino, 2013). Similarly, while the Corsi Block total score is technically discrete rather than continuous, the sufficient range (54–115 in our sample) and distributional properties support parametric analysis when assumptions are met, consistent with standard practice for cognitive test scores.

Effect sizes were calculated using Cohen’s d for parametric tests and rank-biserial correlation for non-parametric tests. Statistical significance was set at *α* = 0.05.

#### 2.3.2 *NavCity* task

To address our central hypothesis on aging-related effects on *NavCity* performance, we fitted linear mixed models (LMMs) with Age Group, Block, and their interaction as fixed effects, while Target and Participant were included as random effects. The model was specified as: Outcome ~ Age_Group * Block + (1|Target) + (1|Participant). Age Group was contrast-coded with YAs as the reference group, and Block was contrast-coded with three levels (Block 1, Block 2, Block 3), generating pairwise comparisons across blocks. LMMs were run for each outcome measure using the *lme4* package (Bates et al., 2014) with *p*-values obtained via the *lmerTest* package (Kuznetsova et al., 2017) in RStudio (Version 2023.06.1).

Target was included in the models as a random effect to account for potential target-specific variation, as our prior work in younger adults identified significant target effects in *NavCity* performance (Bassil et al., 2025). Since our current research question focused on aging-related differences rather than establishing *NavCity* baseline performance, we used a focused model specification that avoided over-parameterization of target-specific age interactions for which there was not a prior hypothesis.

Post-hoc analyses followed a hierarchical approach, beginning with ANOVA tests on fitted LMMs to evaluate the overall significance of main effects (Age Group, Block) and their interaction. Subsequently, planned contrasts were evaluated using the *emmeans* package (Lenth, 2023), which included: (1) between-group comparisons within each block (YA vs. OA for Block 1, Block 2, and Block 3), (2) between-block comparisons within each group (Block 1 vs.2, Block 2 vs. 3, and Block 1 vs.3 for YA and OA), and (3) age group differences in performance across blocks (whether the magnitude of block-to-block improvement differed between YA and OA). This approach focused on interpretable contrasts while avoiding uninformative cross-condition comparisons. Age Group was contrast-coded with Young Adults as the reference group, and Block was coded with Block 1 as the reference level. *p*-values were adjusted using the false discovery rate (FDR) method within each outcome measure to control for multiple comparisons.

#### 2.3.3 *NavCity* allocentric representation assessment (NARA)

NARA scores were calculated using the NARA Scoring Rubric, previously established in younger adults (Bassil et al., 2025). Here, we applied this scoring system to compare this aspect of spatial knowledge recall between younger and older adults.

NARA scores were compared between age groups to assess differences in allocentric knowledge recall. Though both groups had adequate sample sizes (≥30) and equal variances (Levene’s test: *F* = 2.91, *p* = 0.09), a two-sided Mann–Whitney U test was conducted due to non-normal distributions in both groups (Shapiro–Wilk test: YAs W = 0.907, *p* = 0.012; OAs W = 0.900, *p* = 0.008). This test was paired with a rank biserial correlation to calculate effect size.

Associations between NARA scores and each *NavCity* outcome measure were evaluated with non-parametric analyses using Spearman’s rank correlations with significance set at α = 0.05. Fisher’s Z-transformation was used to test whether strength of correlation coefficients differed between age groups. To assess whether NARA scores capture variance beyond chronological age alone, correlations between NARA scores and age were also evaluated using the same statistical parameters. Correlation coefficients were interpreted using Cohen’s conventions for small (r = 0.10), medium (r = 0.30), and large (r = 0.50) effect sizes (Cohen, 1988), though recent work suggests these thresholds may be conservative (Gignac and Szodorai, 2016).

## 3 Results

### 3.1 Demographics, questionnaires, and cognitive tasks

#### 3.1.1 Participant characteristics

The YA and OA groups were similar for several demographic characteristics, including gender distribution (YA: 56.7% women, 43.3% men; OA: 60% women, 40% men; χ^2^ = 0.07, *p* = 0.79, Cramér’s V = 0.03) and handedness (YA: 83.3% right-handed; OA: 86.7% right-handed; Fisher’s exact test: *p* = 1.0). Weekly exercise frequency was also similar between groups (YA: Mdn[IQR] = 3.9[2.63, 6.00], OA: Mdn[IQR] = 4.8[3.00, 7.00]; Mann–Whitney U test: W = 408.5, *p* = 0.54, r = 0.09). Sleep quality, as measured by the PSQI, also did not differ significantly between groups (YA: Mdn[IQR] = 5[4, 6]; OA: Mdn[IQR] = 4.5[2, 7]; Mann–Whitney U test: W = 472, *p* = 0.75, r = −0.05).

However, groups differed significantly in technology experience. YA participants reported more prior VR exposure compared to OA participants (YAs: Mdn [IQR] = 1[0, 2], OAs: Mdn[IQR] = 0[0, 1]; Mann–Whitney U test: W = 621, *p* = 0.005, r = −0.38) and higher weekly video game usage (YA: Mdn[IQR] = 1.5[0, 3.75], OA: Mdn[IQR] = 0[0, 0]; Mann–Whitney U test: W = 643.5, *p <* 0.001, r = −0.43).

Additionally, OA participants reported better navigational confidence on the SBSOD (M = 5.08, SD = 0.93), compared to YA participants (M = 4.35, SD = 1.10; independent samples *t*-test: *t*(58) = −2.79, *p* = 0.007, 95% CI = [−1.26, −0.21], d = −0.72).

YA participants also reported higher baseline sleepiness on the SSS compared to OA participants (YA: Mdn[IQR] = 2[1–2], OA: Mdn *=* 1[1–1.75]; Mann–Whitney U test: W = 639, *p* = 0.002, r = −0.42). However, post-study sleepiness (YA: Mdn [IQR] = 1[1–3]; OA: Mdn[IQR] = 1[1–2]; Mann–Whitney U test: W = 534, *p* = 0.16, r = −0.19) and change in sleepiness (YA: Mdn[IQR] = 0[−0.75–0.75]; OA: Mdn[IQR] = 0[0–0]; Mann–Whitney U test: W = 413.5, *p* = 0.55, r = 0.08) did not differ significantly between groups.

Measures of VR-induced effects or sickness, measured by the SSQ, were not significantly different between groups: at baseline (SSQ_Pre_ YA: Mdn[IQR] = 2[0, 3]; OA: Mdn[IQR] = 1[0, 2.75]; Mann–Whitney U test: W = 494, *p* = 0.51, r = −0.10), after the session (SSQ_Post_ YA Mdn[IQR] = 1.5[0, 4.75]; OA Mdn[IQR] = 1.5[0, 4.75]; Mann–Whitney U test: W = 431, *p* = 0.78, r = 0.04), nor change across the session (ΔSSQ: YA Mdn[IQR] = 0[0, 2.75]; OA Mdn[IQR] = 0[0, 2]; Mann–Whitney U test: W = 461.5, *p* = 0.87, r = −0.03).

#### 3.1.2 Cognitive performance

Groups showed significant aging-related differences in cognitive performance. YAs completed the Trail Making Test A faster (M = 25.9, SD = 4.56) than OAs (M = 34.2, SD = 6.84; independent samples *t*-test: *t*(58) = −5.53, *p <* 0.001, 95% CI = [−11.31, −5.30], d = −1.43). Similarly, YAs completed Trail Making Test B faster (Mdn[IQR] = 35.5[29.68, 38.00]) than OAs (Mdn[IQR] = 48.6[44.55, 65.90]; Mann–Whitney U test: W = 151.5, *p <* 0.001, r = 0.66). The Trail Making Test B-A difference was significantly smaller in YAs (Mdn[IQR] = 8.45[5.13, 14.78]) than OAs (Mdn[IQR] = 17.3[11.60, 23.48]; Mann–Whitney U test: W = 237, p = 0.002, r = 0.47), indicating higher cognitive set shifting performance. YAs also showed better visuospatial working memory on the Corsi Block Test (YA: M = 89.2, SD = 10.4; OA: M = 70.9, SD = 9.83; independent samples *t*-test: *t*(58) = 7.01, *p <* 0.001, 95% CI = [13.10, 23.57], d = 1.81). Individual data points and full results can be found in Supplementary Table S1.

### 3.2 Aging effects on naturalistic navigation performance in *NavCity*

#### 3.2.1 *NavCity* primary outcomes

To examine aging-related differences in navigation performance, we initially focused on our primary *NavCity* outcomes, including speed, distance traveled, and navigation time, which are measures that are frequently used to study fundamental aspects of navigation performance (Ruddle and Lessels, 2006) and are often reported as central measures in meta-analytic studies (Nazareth et al., 2019; Plácido et al., 2022).

**Figure 2:**
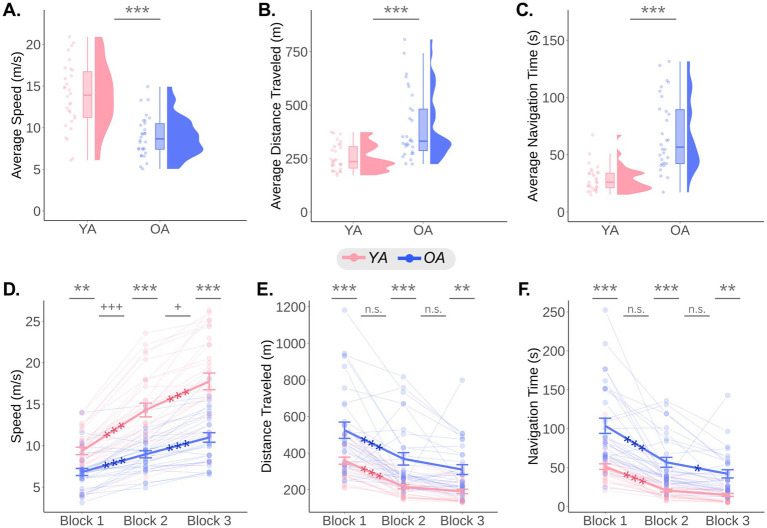
Aging effects on NavCity primary navigation performance outcomes. **(A–C)** Overall *NavCity* performance for primary navigation outcomes (speed, distance traveled, and navigation time) averaged across all exposure blocks. **(D–F)** Change in performance across exposure blocks, with primary outcomes averaged across 8 target buildings per block for each participant. Violin plots **(A–C)** show data distributions with overlaid box plots indicating median and quartiles. Line plots **(D–F)** show individual participant trajectories (colored points connected by lines) with group means ± standard error. Statistical significance: *p < 0.05, **p < 0.01, ***p < 0.001, n.s. = not significant. Horizontal lines with asterisks (*) denote significant between-group comparisons (YA vs. OA). Horizontal lines with plus signs (+) indicate significant group x block interactions (different improvement rates between age groups). Asterisks (*) on trend lines within plot indicate within-group differences between consecutive blocks).

As expected, OAs demonstrated significantly lower overall performance across all primary navigation measures (Figures 2A–C), including slower speed, greater distance traveled, and longer navigation time (*β*_speed_ = 4.89, *β*_dist_ = −146.36, *β*_time_ = −38.58; all p_corr_ < 0.001). These group differences were present at each individual exposure block (Figures 2D–F), with OAs showing slower speeds (*β*_B1_ = 2.55, *β*_B2_ = 5.34, *β*_B3_ = 6.77; all p_corr_ ≤ 0.006), greater distances traveled (*β*_B1_ = −166.69, *β*_B2_ = −152.62, *β*_B3_ = −119.78; all p_corr_ ≤ 0.004), and longer navigation times (*β*_B1_ = −52.43, *β*_B2_ = −36.22, *β*_B3_ = −27.10; all p_corr_ < 0.001) per block, compared to YAs.

When evaluating change in navigation performance across blocks, both age groups showed significant improvement with exposure, but with different patterns across measures (Figures 2D–F). For speed, both age groups improved significantly across all consecutive blocks (YA: *β*_B1 − 2_ = −4.93, *β*_B2-3_ = −3.45; OA: *β*_B1 − 2_ = −2.14, *β*_B2-3_ = −2.03; all p_corr_ < 0.001). However, YAs showed a significantly larger rate of improvement than OAs across all block comparisons (YA-OA: *β*_B1-2_ = −2.80, *β*_B2-3_ = −1.42; both p_corr_ ≤ 0.011) (Figure 2D). For distance, both groups improved significantly from Block 1 to Block 2 (YA *β* = 142.62, OA *β* = 156.69; both p_corr_ < 0.001), but neither group showed further improvement from Block 2 to Block 3 (p_corr_ > 0.05), with no difference in performance change between age groups across blocks (all p_corr_ > 0.05) (Figure 2E). Navigation time showed the most complex pattern, where both groups improved from Block 1 to Block 2 (YA *β* = 30.67, OA *β* = 46.88; both p_corr_ < 0.001), but only OAs demonstrated additional significant improvement from Block 2 to 3 (*β* = 14.79, p_corr_ = 0.017), with no significant group difference in performance change between exposure blocks (all p_corr_ > 0.05) (Figure 2F).

#### 3.2.2 *NavCity* secondary outcomes

We then examined secondary *NavCity* outcome measures that provide additional behavioral insights to navigation performance, including average dwell duration, teleportation count, and average teleportation distance.

**Figure 3:**
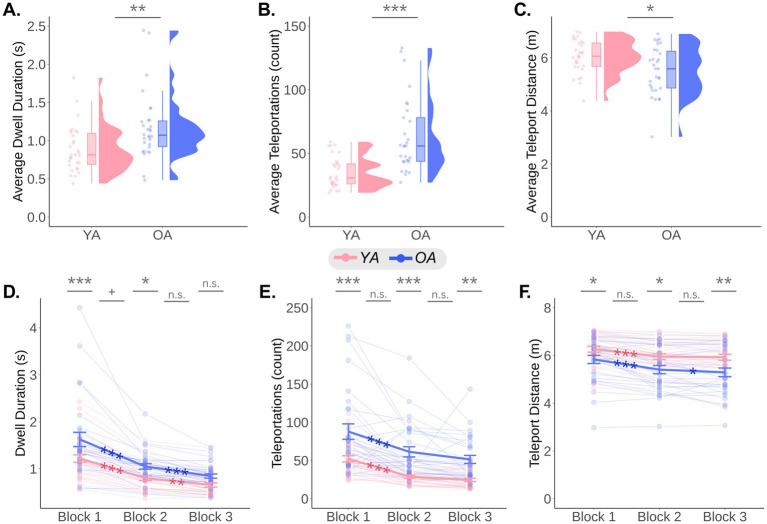
Aging effects on NavCity secondary navigation performance outcomes. **(A–C)** Overall *NavCity* performance for secondary navigation outcomes (dwell duration, teleportation count, and teleport distance) averaged across all exposure blocks. **(D–F)** Change in performance across exposure blocks, with secondary outcomes averaged across 8 target buildings per block for each participant. Violin plots **(A–C)** show data distributions with overlaid box plots indicating median and quartiles. Line plots **(D–F)** show individual participant trajectories (colored points connected by lines) with group means ± standard error. Statistical significance: *p < 0.05, **p < 0.01, ***p < 0.001, n.s. = not significant. Horizontal lines with asterisks (*) denote significant between-group comparisons (YA vs. OA). Horizontal lines with plus signs (+) indicate significant group x block interactions (different improvement rates between age groups). Asterisks (*) on trend lines within plot indicate within-group differences between consecutive blocks).

Consistent with the primary analyses, OAs demonstrated significantly lower performance on secondary outcomes (Figures 3A–C), showing longer average dwell durations, higher teleportation counts, and shorter teleportation distances compared to YAs (*β*_dwell_ = −0.28, *β*_t-count_ = −31.64, *β*_t-dist_ = 0.54; all p_corr_ ≤ 0.01) (Figures 3A–C). However, the pattern of group differences varied across individual blocks (Figures 3D–F). For dwell duration, OAs showed longer dwell durations than YAs in Block 1 and Block 2 (*β*_B1_ = −0.41, *β*_B2_ = −0.25; both p_corr_ ≤ 0.022), but similar dwell durations in Block 3 (p_corr_ > 0.05). Otherwise, OAs consistently performed differently across all block comparisons, with more teleportations (*β*_B1_ = −35.69, *β*_B2_ = −32.45, *β*_B3_ = −26.78; all p_corr_ ≤ 0.002), and shorter average teleportation distances (*β*_B1_ = 0.43, *β*_B2_ = 0.54, *β*_B3_ = 0.64; all p_corr_ ≤ 0.042) than YAs.

When measuring change in navigation performance across blocks, both age groups showed significant improvement with exposure, similar to patterns in primary measures (Figures 3D–F). Both age groups reduced dwell duration across all consecutive blocks (YA: *β*_B1-2_ = 0.42, *β*_B2-3_ = 0.15; OA: *β*_B1-2_ = 0.57, *β*_B2-3_ = 0.21; all p_corr_ ≤ 0.003), with YAs showing significantly greater reductions than OAs from Block 1 to 2 (*β* = −0.16, p_corr_ = 0.038) but not Block 2 to 3 (p_corr_ > 0.05) (Figure 3D). Both groups reduced the number of teleportations from Block 1 to 2 (YA *β* = 23.25, OA *β* = 26.49; both p_corr_ < 0.001) but neither group showed further improvement from Block 2 to 3 (all p_corr_ > 0.05) with no difference in learning rate between groups (all p_corr_ > 0.05) (Figure 3E). Teleportation distances showed the most complex pattern, where both groups reduced distance from Block 1 to 2 (YA: *β* = 0.31, OA: *β* = 0.43; both p_corr_ < 0.001), but only OAs continued to reduce distance from Block 2 to 3 (*β* = 0.11, p_corr_ = 0.017), with no significant group difference in performance change between blocks (all p_corr_ > 0.05) (Figure 3F).

### 3.3 Aging effects on allocentric spatial knowledge recall tied to *NavCity*

We next examined aging-related differences in allocentric spatial knowledge recall using NARA scores and explored how this recall related to navigation performance across both age groups.

#### 3.3.1 Aging-related effects on NARA performance

**Figure 4:**
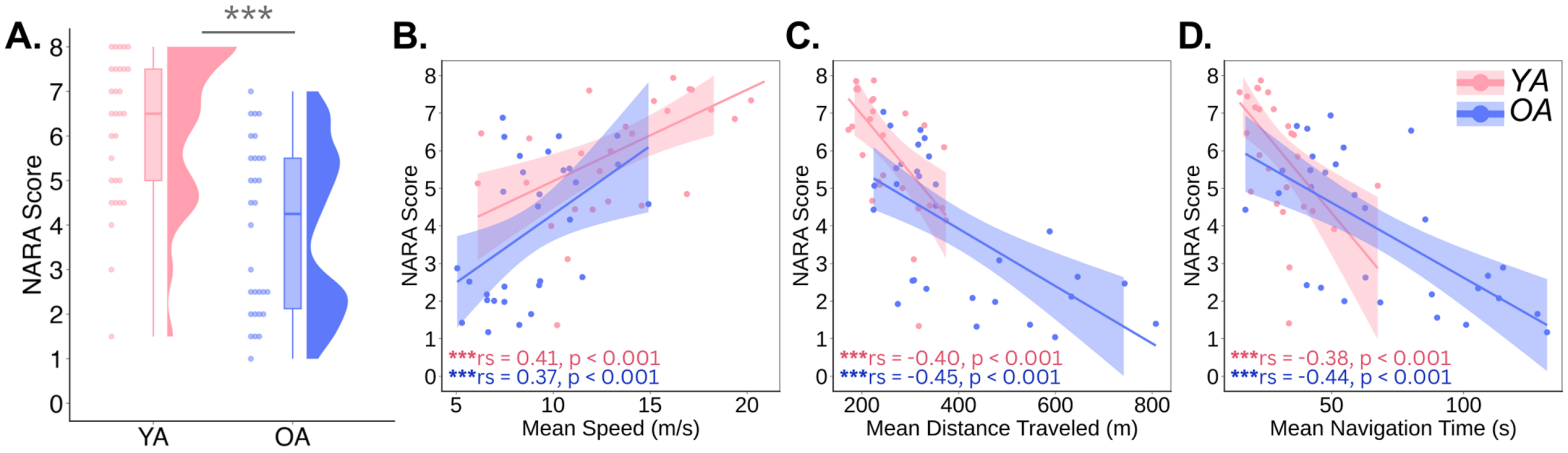
NARA scores and correlations with NavCity performance. **(A)** *NavCity* allocentric representation assessment (NARA) scores, a measure of allocentric knowledge recall, were lower for the older adult (OA) group compared to the younger adult (YA) group (*** *p <* 0.001). **(B–D)** Correlations between individual participants’ NARA scores and navigation metrics, showing relationships with mean speed **(B)**, distance traveled **(C)**, and navigation time **(D)**. Violin plots show data distributions with overlaid box plots indicating median and quartiles. Scatter plots show Spearman’s rank correlations with regression lines and 95% confidence intervals for each age group.

A Mann–Whitney U test revealed a significant difference in NARA scores between age groups (W = 179.5, n_YA_ = 30, n_OA_ = 30, *p <* 0.001), with a large effect size (r = 0.60) (Figure 4A). The OA group demonstrated significantly lower NARA scores than YA, with median scores of 4.25 (IQR_OA_ = 2–5.5) and 6.50 (IQR_YA_ = 5–7.5) respectively. There was no significant correlation between NARA score and biological age within YAs (r_s_ = 0.01, *p* = 0.96) or OAs (r_s_ = −0.12, *p* = 0.539).

Visual inspection of NARA scores revealed a potential bimodal distribution in the OA group (Figure 4A). This observation was confirmed by Hartigan’s dip test (Hartigan and Hartigan, 1985), implemented in R using the *diptest* package (Maechler and Ringach, 2025), which indicated that NARA scores for the OA group deviated significantly from unimodality (D = 0.10, *p* = 0.013), suggesting the presence of at least two distinct modes in the older adult distribution. To determine the optimal cutoff for the bimodal NARA distribution, we used gap detection analysis, implemented using custom R code, which identifies the largest gap between consecutive scores and places the cutoff at the midpoint of this separation. Specifically, NARA scores were sorted in ascending order, pairwise gaps between consecutive scores were computed using the *diff()* function, the position of the largest gap was identified, and the cutoff was placed at the midpoint of this largest gap. This method avoids arbitrary thresholding and places the cutoff where the data naturally separates into two distinct groups. The largest gap occurred between consecutive NARA scores of 3.0 and 4.0 (gap size = 1.0), resulting in an optimal cutoff of 3.5. This cutoff resulted in 14 participants in the “OA_Low_” group (NARA < 3.5) and 16 participants in the “OA_High_” group (NARA ≥ 3.5).

Within the YA group, NARA scores showed no bimodality (Hartigan’s dip test: D = 0.083, *p* = 0.086) but departed from normality (Shapiro–Wilk test: W = 0.907, *p* = 0.012) due to negative skewness (−0.799), with kurtosis near zero (−0.03). Nearly all YAs scored above the NARA threshold used to define the OA group (NARA ≥ 3.5), with two exceptions (S01, S23). These participants were not statistical outliers (IQR-based method) on any *NavCity* outcome measures or most cognitive assessments. However, S23 showed outlier values for PSQI and Trails B, showing significantly lower sleep quality (PSQI = 12) and higher Trails B completion time (17.19 s).

#### 3.3.2 Associations between *NavCity* primary outcomes and NARA performance

Correlation analyses revealed significant associations between all mean *NavCity* primary outcomes and NARA scores were statistically significant for YAs, including speed (r_s_ = 0.41, *p <* 0.001), distance traveled (r_s_ = −0.40, *p <* 0.001), and navigation time (r_s_ = −0.38, *p <* 0.001). The same relationships were significant for OAs, including speed (r_s_ = 0.37, *p <* 0.001), distance traveled (r_s_ = −0.45, *p <* 0.001), and navigation time (r_s_ = −0.44, *p <* 0.001) (Figures 4B–D). All correlations demonstrated medium effect sizes by Cohen’s conventions and correlations did not differ between age groups for speed (Z = 0.34, *p* = 0.733), distance (Z = 0.44, *p* = 0.657), nor navigation time (Z = 0.45, *p* = 0.655). Additionally, NARA scores were not significantly associated with chronological age for YAs (r_s_ = 0.01, p = 0.96) nor OAs (r_s_ = −0.117, p = 0.539).

### 3.4 *NavCity* performance in NARA-defined subgroups

Given the bimodal distribution of NARA scores within the OA group, we subdivided the OA group and created 3 NARA-defined subgroups: YA, OA_High_, OA_Low_. Within NARA, OA_Low_ (*n =* 14) had scores ranging from 1 to 3 (M = 2.04 ± 0.54), OA_High_ (*n =* 16) with scores ranging from 4.0 to 7.0 (M = 5.53 ± 0.85), and YAs showed scores ranging from 1.5 to 8 (M = 6.12 ± 1.66). There was no significant correlation between NARA scores and biological age within OA_High_ (r_S_ = 0.32, *p* = 0.229) or OA_Low_ (r_s_ = −0.002, *p* = 0.994).

To confirm that the NARA-based subgroups reflected differences in spatial ability rather than demographic characteristics or general cognitive function, we compared OA_High_ and OA_Low_ groups across all questionnaire and task outcomes collected, including demographics (gender, handedness), lifestyle factors (VR experience, video game usage, exercise frequency, PSQI), cognitive function (SBSOD, Trails Making A and B, Corsi Block), and pre- and post-session measures (SSS, SSQ). No significant differences emerged between the OA_High_ and OA_Low_ subgroups on any of these measures (all p_corr_ > 0.05).

We next examined whether this subdivision into OA_High_ and OA_Low_ performers corresponded to meaningful differences in navigation behavior during *NavCity*. If allocentric spatial knowledge recall ability contributes to the heterogeneity we observed in NARA scores, we would expect the OA_High_ group to demonstrate navigation performance that more closely resembles the YA group while the OA_Low_ group may show more pronounced navigation difficulties. To test this expectation, we re-analyzed all *NavCity* outcome measures using 3 groups: YA (*n =* 30), OA_High_ (NARA ≥ 3.5, *n =* 16), and OA_Low_ (NARA < 3.5, *n =* 14), examining both overall performance differences and improvements across exposure blocks. Statistical analyses were identical to those previously described, except that comparisons now involved two NARA-defined groups within the OA cohort, in addition to YAs. Age Group was contrast coded with 3 levels (YA, OA_High_, OA_Low_).

#### 3.4.1 *NavCity* primary outcomes

**Figure 5:**
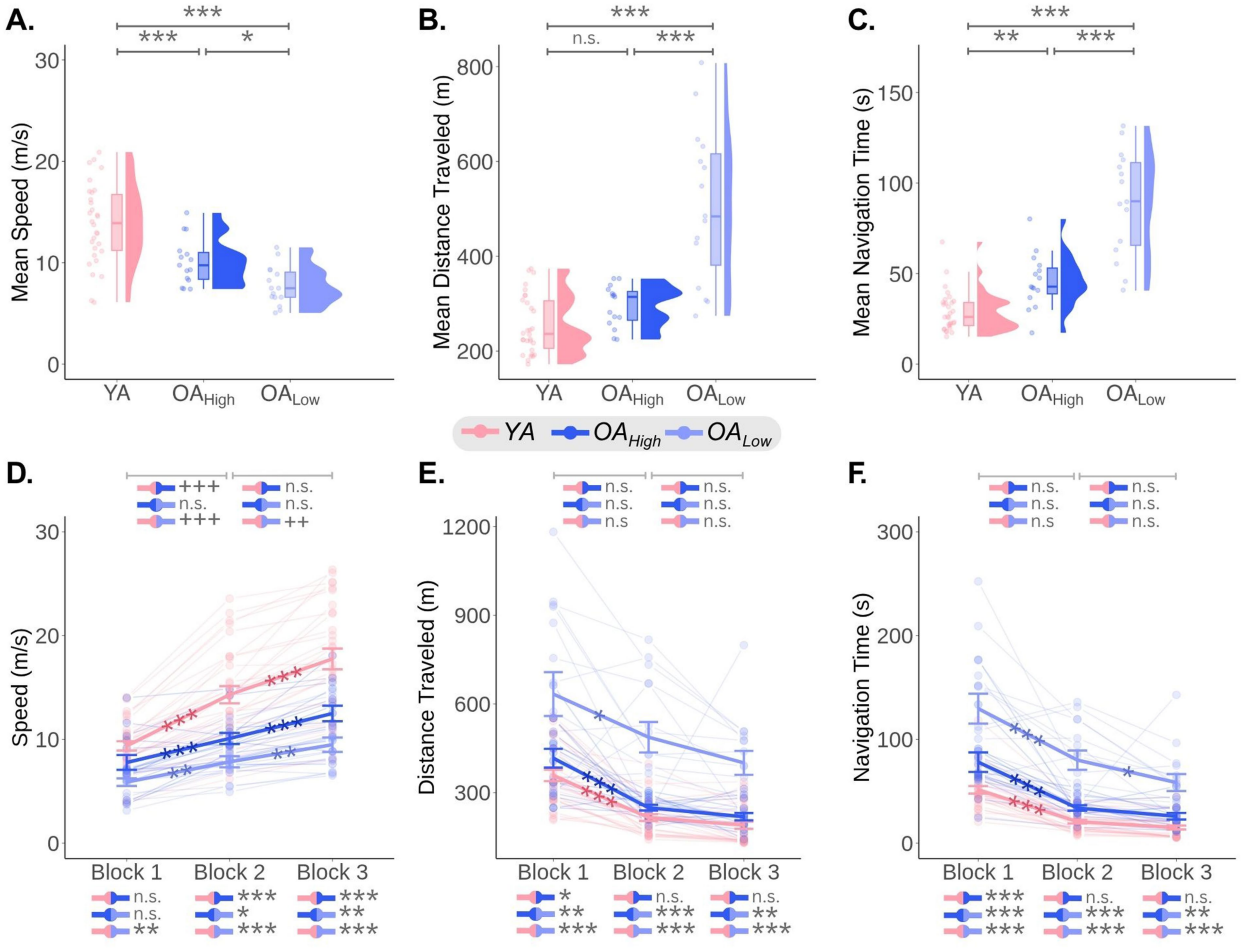
NARA-defined subgroup performance for NavCity primary outcomes. **(A–C)** Overall *NavCity* performance by NARA-defined subgroups for primary navigation outcomes (speed, distance traveled, and navigation time) averaged across all exposure blocks. (D–F) Block-specific performance showing change in performance across blocks, with primary outcomes averaged across 8 target buildings per block for each participant. Violin plots **(A–C)** show data distributions with overlaid box plots indicating median and quartiles. Line plots **(D–F)** show individual participant trajectories (colored points connected by lines) with group means ± standard error. Statistical significance: * *p <* 0.05, ** *p <* 0.01, *** *p <* 0.001, n.s. = not significant. For mean *NavCity* outcomes **(A–C)**, horizontal lines with asterisks denote between-group comparisons (YA vs. OA High, YA vs. OA Low, OA High vs. OA Low). For block-specific outcomes **(D–F)**, icons below plots indicate within-block comparisons between NARA subgroups. Asterisks on trend lines indicate within-group differences between consecutive blocks. Plus signs above plots indicate significant group × block interactions (different learning rates between age groups).

For overall navigation performance, we examined primary *NavCity* outcomes averaged across blocks across the 3 NARA-defined subgroups. Speed and navigation time followed a clear pattern: YA > OA_High_ > OA_Low_ (Figures 5A,C), with all pairwise comparisons reaching significance for speed (YA-OA_High_ *β* = 3.64, OA_High_-OA_Low_ *β* = 2.66, YA-OA_Low_ *β* = 6.30; all p_corr_ ≤ 0.025) and navigation time (YA-OA_High_ *β* = −19.35, OA_High_-OA_Low_ *β* = −41.22, YA-OA_Low_ *β* = −60.57; all p_corr_ ≤ 0.002). Distance traveled showed a different pattern, with YA and OA_High_ performing similarly (p_corr_ > 0.05), while both YA and OA_High_ groups traveled significantly shorter distances than OA_Low_ (OA_High_-OA_Low_ *β* = −188.31, YA-OA_Low_ *β* = −246.80; both p_corr_ < 0.001) (Figure 5B).

Block-specific analyses revealed how group differences evolved across exposure (Figures 5D–F). Speed showed progressive differentiation across blocks, with between-group differences becoming more pronounced with exposure (Figure 5D). In Block 1, OA_Low_ performed significantly slower than YA (*β* = 3.53, p_corr_ = 0.006), while other comparisons were not significant (p_corr_ > 0.05). However, by Blocks 2 and 3, all pairwise comparisons reached significance with the difference across groups becoming increasingly pronounced (Block 2: YA-OA_High_ *β* = 4.12, OA_High_-OA_Low_ *β* = 2.63, YA-OA_Low_ *β* = 6.75; Block 3: YA-OA_High_ *β* = 5.13, OA_High_-OA_Low_ *β* = 3.51, YA-OA_Low_ *β* = 8.64; all p_corr_ ≤ 0.038).

Distance traveled and navigation time demonstrated similar patterns to each other, with YA and OA_High_ converging to similar performance in later blocks (Figures 5E,F). For Block 1, all groups differed significantly across blocks for distance traveled (YA-OA_High_ *β* = −91.28, OA_High_-OA_Low_ *β* = −161.60, YA-OA_Low_ *β* = −252.88; all p_corr_ ≤ 0.043) and navigation time (YA-OA_High_ *β* = −32.75, OA_High_-OA_Low_ *β* = −42.17, YA-OA_Low_ *β* = −74.92; all p_corr_ < 0.001). However, in Blocks 2 and 3, YA and OA_High_ demonstrated similar performance (all p_corr_ > 0.05), while both groups continued to outperform OA_Low_. Specifically, OA_Low_ showed longer distances (Block 2: OA_High_-OA_Low_ *β* = −231.06, YA-OA_Low_ *β* = −275.85; Block 3: OA_High_-OA_Low_ *β* = −172.28, YA-OA_Low_ *β* = −211.66; all p_corr_ ≤ 0.002), as well as longer navigation times (Block 2: OA_High_-OA_Low_ *β* = −47.79, YA-OA_Low_ *β* = −61.71; Block 3: OA_High_-OA_Low_ *β* = −33.71, YA-OA_Low_ *β* = −45.08; all p_corr_ ≤ 0.001), compared to other groups.

While all groups showed significant performance improvement across exposure blocks, speed was the only primary outcome in which all groups improved between all block comparisons (YA *β*_B1-2_ = −4.93, *β*_B2-3_ = −3.45; OA_High_ *β*_B1-2_ = −2.51, *β*_B2-3_ = −2.44; OA_Low_ *β*_B1-2_ = −1.72, *β*_B2-3_ = −1.56; all p_corr_ ≤ 0.005) (Figure 5D). Furthermore, speed was the only primary outcome in which groups differed significantly in their rate of improvement across blocks. YA showed significantly larger increases in speed than both OA subgroups from Block 1 to 2 (YA-OA_High_ *β* = −2.43, YA-OA_Low_ *β* = −3.22; both p_corr_ < 0.001) and compared to OA_Low_ from Block 2 to 3 (*β* = −1.89, p_corr_ = 0.007). For other primary outcomes, all groups improved significantly from Block 1 to 2, for distance traveled (YA *β* = 142.62, OA_High_ *β* = 189.10, OA_Low_ *β* = 119.64; all p_corr_ ≤ 0.019) and navigation time (YA *β* = 30.68, OA_High_ *β* = 49.50, OA_Low_ *β* = 43.88; all p_corr_ < 0.001) (Figures 5E,F). Only OA_Low_ continued to reduce navigation time from Block 2 to 3 (*β* = 22.29, p_corr_ = 0.014). No significant differences in improvement rates were observed between groups for either outcome (all p_corr_ > 0.05).

#### 3.4.2 *NavCity* secondary outcomes

We next examined secondary navigation outcomes across the 3 NARA-defined groups to gain deeper insight into the behavioral strategies underlying navigation performance differences.

**Figure 6:**
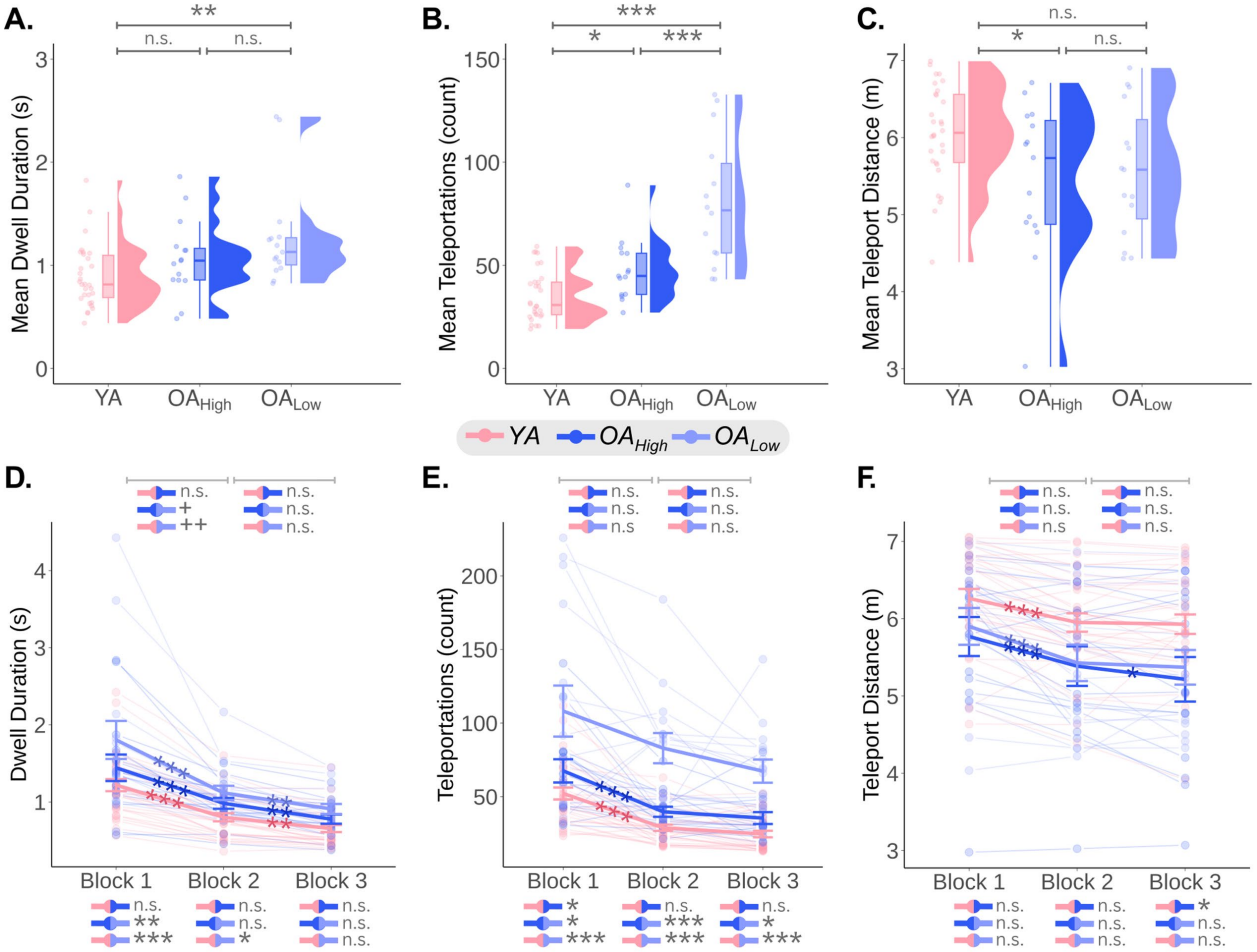
NARA-defined subgroup performance for NavCity secondary outcomes. **(A–C)** Overall *NavCity* performance by NARA-defined subgroups for secondary navigation outcomes (mean dwell duration, teleportation count, and mean teleportation distance) averaged across all exposure blocks. **(D–F)** Block-specific performance showing learning trajectories, with primary outcomes averaged across 8 target buildings per block for each participant. Violin plots **(A–C)** show data distributions with overlaid box plots indicating median and quartiles. Line plots **(D–F)** show individual participant trajectories (colored points connected by lines) with group means ± standard error. Statistical significance: * *p <* 0.05, ** *p <* 0.01, *** *p <* 0.001, n.s. = not significant. For mean *NavCity* outcomes **(A–C)**, horizontal lines with asterisks denote between-group comparisons (YA vs. OA High, YA vs. OA Low, OA High vs. OA Low). For block-specific outcomes **(D–F)**, icons below plots indicate within-block comparisons between NARA subgroups. Asterisks on trend lines indicate within-group differences between consecutive blocks. Plus signs above plots indicate significant group × block interactions (different learning rates between age groups).

When averaged across blocks, secondary outcomes showed distinct patterns (Figures 6A–C). For dwell duration, only YA and OA_Low_ groups differed significantly, with YA showing shorter dwell durations (*β* = −0.42, p_corr_ = 0.003), while OA_High_ performed similarly to both groups (p_corr_ > 0.05). For teleportations, all groups differed significantly following the established hierarchy (YA > OA_High_ > OA_Low_), with YA performing fewer teleportations than OA_High_ (*β* = −17.07, p_corr_ = 0.022), OA_High_ fewer than OA_Low_ (*β* = −31.22, p_corr_ < 0.001), and YA fewer than OA_Low_ (*β* = −48.29, p_corr_ < 0.001). For teleportation distance, only YA and OA_High_ groups differed significantly, with YA demonstrating longer distances (*β* = 0.63, p_corr_ = 0.035), while OA_Low_ performed similarly to both groups (p_corr_ > 0.05). However, a sensitivity analysis excluding one OA_High_ participant with a markedly low mean teleportation distance (Figure 6C) rendered the group-level effect non-significant, indicating the overall YA vs. OA_High_ difference is influenced by this participant (without outlier: *β* = 0.47, pcorr = 0.111), though Block 3 difference remained significant (*β* = 0.61, p_corr_ = 0.035).

Block-specific analyses revealed evolving group differences across exposure (Figures 6D–F). Dwell duration showed converging group performance across blocks (Figure 6D). Block 1 revealed significant differences between OA_High_-OA_Low_ (*β* = −0.43, p_corr_ = 0.008) and YA-OA_Low_ (*β* = −0.63, p_corr_ < 0.001), while YA and OA_High_ performed similarly (p_corr_ > 0.05). By Block 2, only the YA-OA_Low_ difference remained significant (*β* = −0.34, p_corr_ = 0.036), and Block 3 showed no significant group differences (all *p* > 0.05).

Teleportations demonstrated persistent group differences across most blocks, with YA and OA_High_ converging to similar performance after Block 1. Block 1 showed all groups differed significantly (YA-OA_High_ *β* = −24.13, OA_High_-OA_Low_ *β* = −24.78, YA-OA_Low_ *β* = −48.90; all p_corr_ ≤ 0.03), following a similar performance hierarchy (YA > OA_High_ > OA_Low_), with fewer teleportations, given the fixed set of target destinations, reflecting shorter overall distances traveled and thus more efficient route selection. In Blocks 2 and 3, YA and OA_High_ no longer differed significantly (p_corr_ > 0.05), while both groups continued to outperform OA_Low_ (Block 2: OA_High_-OA_Low_ *β* = −40.09, YA-OA_Low_ *β* = −53.83; Block 3: OA_High_-OA_Low_ *β* = −28.79, YA-OA_Low_ *β* = −42.14; all p_corr_ ≤ 0.018) (Figure 6E). Teleportation distance showed few group differences across blocks, with only YA demonstrating longer distances than OA_High_ in Block 3 (*β* = 0.74, p_corr_ = 0.011) (Figure 6F).

All groups showed significant improvement in secondary measures across blocks, but with different patterns across measures (Figures 6D–F). Dwell duration was the only secondary outcome showing continuous improvement across all blocks for all groups (YA *β*_B1-2_ = 0.42, *β*_B2-3_ = 0.15; OA_High_ *β*_B1-2_ = 0.45, *β*_B2-3_ = 0.21; OA_Low_ *β*_B1-2_ = 0.71, *β*_B2-3_ = 0.20; all p_corr_ ≤ 0.005). Groups also showed different improvement rates for dwell duration from Block 1 to 2, with OA_Low_ demonstrating greater reductions in duration than both YA (*β* = −0.30, p_corr_ = 0.001) and OA_High_ (*β* = −0.26, p_corr_ = 0.015), while no differences were observed from Block 2 to 3 (p_corr_ > 0.05) (Figure 6D). For teleportations, significant improvement from Block 1 to 2 was observed only in YA (*β* = 23.25, p_corr_ < 0.001) and OA_High_ (*β* = 33.63, p_corr_ < 0.001), while OA_Low_ showed no improvement across blocks. No differences in the change in teleportation count were observed between groups (all p_corr_ > 0.05) (Figure 6E). Lastly, teleportation distance showed changes primarily from Block 1 to 2 across all groups (YA *β* = 0.31, OA_High_ *β* = 0.37, OA_Low_ *β* = 0.49; all p_corr_ < 0.001), with only OA_High_ continuing to reduce teleportation difference from Block 2 to 3 (*β* = 0.16, p_corr_ = 0.014), but no significant differences in change in teleportation distances between groups were observed (all p_corr_ > 0.05) (Figure 6F).

#### 3.4.3 Associations between *NavCity* primary outcomes and NARA performance within OA subgroups

To address whether the *NavCity*-NARA relationship differs between higher- and lower-performing older adults, we conducted within-subgroup Spearman correlations between NARA scores and each primary *NavCity* outcome (Supplementary Figure S1), paralleling the analyses reported in Section 3.3.2 for the full OA group. All correlations for OA_High_ and OA_Low_ subgroups between NARA and each primary outcome were non-significant (OA_High_: speed r_s_ = −0.34, distance r_s_ = −0.16, navigation time r_s_ = −0.06, all *p* ≥ 0.20; OA_Low_: speed r_s_ = 0.09, distance r_s_ = −0.22, navigation time r_s_ = −0.33, all *p* ≥ 0.24).

## 4 Discussion

The present study investigated aging-related differences in navigation performance and within-session improvement within an immersive virtual reality environment across repeated exposures. Consistent with our hypotheses, older adults demonstrated significantly lower navigation performance than younger adults across all primary outcome measures. Importantly, both age groups showed similar rates of improvement across repeated exposure blocks for navigation time and distance traveled, supporting our hypothesis that healthy older adults retain the capacity for navigation performance improvement despite baseline performance deficits. However, contrary to expectations of uniform improvement, speed emerged as the sole measure showing divergent learning trajectories between age groups, with older adults demonstrating persistently slower rates of improvement across all three VR environment exposures.

Beyond our original hypotheses, a key novel finding emerged showing that individual differences in allocentric spatial knowledge, as measured by the NARA, revealed substantial heterogeneity within the older adult population. When subdivided by NARA performance, higher-performing older adults achieved navigation efficiency comparable to younger adults on spatial measures (distance traveled), despite persistent aging-related differences in temporal measures (speed, navigation time). This stratification demonstrates that cognitive map formation ability—the capacity to transform first-person navigation experiences into third-person survey knowledge—may account for substantial variance in older adult navigation performance to identify a subset of older adults who maintain spatial processing capabilities comparable to younger adults.

### 4.1 Aging-related effects on navigation performance

As expected, older adults demonstrated consistently lower overall navigation performance than younger adults across all three primary outcome measures (Figures 2A–C), aligning with prior work showing aging-related deficits in wayfinding behavior during active spatial navigation (Klencklen et al., 2012; Lester et al., 2017; Lithfous et al., 2013; Moffat, 2009; Moffat and Resnick, 2002). Performance differences between age groups also persisted for each individual exposure block, with older adults continuing to show significantly lower performance than younger adults even at the final exposure (Figures 2D–F). Prior work has demonstrated that age groups may not fully converge even with extensive training (Baltes and Kliegl, 1992; Head and Isom, 2010; Lövdén et al., 2012; Moffat and Resnick, 2002; Nemmi et al., 2017); however, methodological factors in such tasks (i.e., passive presentation modes, non-immersive 2D displays) may have limited the convergence potential observed in these studies. Notably, active navigation produces larger memory enhancements in older adults than in younger adults compared to passive navigation (Meade et al., 2019), and immersive virtual reality attenuates aging-related navigation differences compared to non-immersive desktop environments (Hill et al., 2024), which suggests that active, immersive paradigms may be particularly effective at narrowing aging-related performance gaps. Given that our task employed an immersive, active VR environment, extended exposure through either additional blocks within sessions or repeated sessions across days may reveal whether aging-related performance gaps can narrow with sufficient practice in more ecologically-relevant contexts.

The consistent pattern of aging-related impairment across all primary measures suggests that multiple aspects of spatial navigation are affected by aging. Lower efficiency on time-dependent measures exhibited by older adults, such as longer navigation times and slower speeds, may be reflective of decreased cognitive processing speed with advancing age, which has been robustly shown as a strong predictor of performance across cognitive tasks in older adults and forms the foundation of a major hypothesis for aging-related cognitive decline (Eckert et al., 2010). Time-dependent measures could also be affected by aging-related decreases in spatial information processing efficiency (Meng et al., 2019), including slower encoding of landmark configurations and delayed retrieval of spatial memories. The combination of general processing speed decline and domain-specific spatial processing deficits may have multiplicative effects on time-dependent navigation outcomes.

Additionally, lower route accuracy reflected by distance-dependent measures in older adults, such as longer distances traveled, may reflect multiple spatial processing impairments. For instance, path integration—the ability to continuously update one’s position relative to a starting point through self-motion cues—declines with age (Adamo et al., 2012; Mahmood et al., 2009), potentially leading to accumulating spatial error and suboptimal route choices. Impairments in cognitive map formation may also prevent older adults from recognizing spatial shortcuts or more efficient alternative routes (Hartmeyer et al., 2017), potentially contributing to the increased distances traveled by older adults. As distance traveled is widely recognized as a primary measure of spatial accuracy (i.e., directly quantifying whether participants know the correct route to targets), deficits on this measure may indicate fundamental impairments in spatial knowledge rather than simply slower or more cautious execution of otherwise accurate routes.

Among secondary outcome measures, older adults also demonstrated lower overall navigation performance than younger adults (Figures 3A–C), with mixed results per individual block exposure depending on outcome. First, dwell time showed a notable pattern: although older adults exhibited longer dwell times than younger adults at first, this difference diminished across repeated exposures, with both groups showing similar performance by the last exposure block (Figure 3D). This convergence suggests that older adults’ dwell behavior, potentially reflecting spatial decision-making efficiency, approached that of younger adults with practice, despite persistent aging-related differences in primary navigation outcomes. While previous work has shown older adults spend more time fixating on landmarks during spatial encoding (Segen et al., 2021), this convergence in dwell time with practice across repeated exposures appears to be a novel finding, highlighting that not all aging-related behavioral differences in virtual navigation remain stable across exposure blocks.

Additionally, teleport distance revealed consistent aging-related differences: older adults used significantly shorter teleport distances than younger adults in all exposure blocks, with the effect size growing larger across blocks (*β*_B1_ = 0.43, *β*_B2_ = 0.54, *β*_B3_ = 0.64; Figure 3F). This pattern suggests that aging-related differences in use of the teleportation interface are present throughout the task, with older adults consistently teleporting to locations closer to their body position (i.e., smaller teleport distances), compared to younger adults. One possible interpretation of this behavioral shift is that it reflects older adults’ well-documented tendency to preferentially attend to proximal rather than distal environmental features during spatial navigation (Moffat and Resnick, 2002; Rodgers et al., 2012). Under this interpretation, a bias toward local, proximal cues (i.e., beacon-based cues), reflected in shorter teleport distances, may represent an adaptive compensatory strategy (Tomaszewski Farias et al., 2018) that reduces cognitive load by breaking complex navigation into smaller, more manageable steps (Thalmann et al., 2019). However, when older adults were further subdivided by NARA performance, this effect was more modest: only the YA vs. OA_High_ comparison reached significance, and a sensitivity analysis showed this overall effect was driven in part by one OA_High_ participant (see Results). Notably, the YA vs. OA_High_ difference remained significant in Block 3, which does not map to the idea of deficit-driven proximal cue bias, given that OA_High_ represents the higher-performing subgroup. Since the characterization of subgroup-level locomotion patterns is beyond the scope of the current work, additional work with larger samples will be needed to determine whether teleport distances reflect proximal cue bias or individual differences in virtual locomotion style.

### 4.2 Improvement in navigation performance with repeated exposure

Despite baseline differences in navigation performance, both age groups showed similar rates of improvement for navigation time and distance traveled with repeated exposure across blocks. This parallel trajectory of improvement demonstrates preserved capacity to improve spatial performance in healthy older adults, consistent with prior work showing that groups across the age spectrum improve similarly in novel environments (Gazova et al., 2013; Head and Isom, 2010; Lövdén et al., 2012; Moffat et al., 2001; Nemmi et al., 2017). This pattern distinguishes healthy aging from early-stage Alzheimer’s disease, where within-session performance improvement on spatial tasks is fundamentally impaired (Gazova et al., 2012; Hort et al., 2007; Laczó et al., 2009, 2011).

However, speed emerged as the only outcome showing both consistent improvement across exposures with divergent improvement trajectories between age groups. Younger adults showed steeper improvements in speed across all three exposure blocks, while older adults improved at a significantly slower rate. This pattern likely reflects the nature of speed as a composite measure that integrates multiple components of navigation performance, including increased certainty in navigation decisions, familiarity with VR controls, and efficiency of movement planning and execution within the virtual environment. The divergent improvement trajectories in speed suggest that, while older adults can learn to optimize their routes (as evidenced by equivalent improvement rates in distance traveled) or their overall task completion (as evidenced by similar improvement rates in navigation time), the rate (or speed) at which they can execute these improved navigation strategies and traverse the virtual environment remain constrained by aging-related factors. Since speed appears to be responsive and sensitive to improvement across repeated exposures in older adults, this suggests that speed could be used as a potential measure to detect and subsequently improve navigation deficits.

Collectively, these findings demonstrate that healthy older adults retain the ability for significant navigation performance improvement in immersive VR environments, with rates of improvement comparable to younger adults for most navigation outcomes despite persistent baseline differences. The selective patterns of behavioral change across measures—convergence in dwell time, divergence in teleport distance, and stable disparities in speed, distance, and time —reveal that aging does not appear to uniformly affect all components of virtual navigation behavior.

### 4.3 Aging effects on cognitive map formation

The NARA task assessed participants’ ability to form cognitive maps by requiring them to construct a top-down, survey-perspective map of the *NavCity* environment based solely on their first-person navigation experiences. As hypothesized, older adults demonstrated significantly lower NARA scores compared to younger adults, with a large effect size (Figure 4A), indicating an aging-related impairment in transforming first-person navigation experiences into allocentric, survey-level spatial knowledge, or ‘cognitive maps.’ This finding aligns with prior work documenting aging-related declines in allocentric spatial processing and cognitive map formation (Harris et al., 2012; Head and Isom, 2010; Iaria et al., 2009; Moffat, 2009; Moffat et al., 2006; Moffat and Resnick, 2002).

The observed aging-related deficit in NARA performance has important theoretical implications, as cognitive maps are considered fundamental to efficient navigation (O’Keefe and Nadel, 1978; Tolman, 1948). The ability to form such representations requires integrating spatial information encountered sequentially during navigation into a unified, coherent spatial framework (Byrne et al., 2007). Lower NARA scores in older adults suggest that this integration process is compromised with aging, potentially reflecting changes in hippocampal function and its interactions with broader medial temporal lobe and posterior parietal networks known to support allocentric spatial processing (Calton and Taube, 2009; Ekstrom et al., 2014; Maguire et al., 1998; Mitchell et al., 2018; Sherrill et al., 2013).

An additional interpretation is that lower NARA scores in older adults reflect difficulty transforming spatial information *between* perspectives, rather than (or in addition to) a deficit in constructing the underlying allocentric representation itself. The NARA requires participants to translate first-person, viewpoint-dependent experience accumulated during *NavCity* into a third-person, top-down representation, such that successful performance depends on flexibly accessing spatial information across the egocentric-to-allocentric continuum (Ekstrom et al., 2017; Starrett and Ekstrom, 2018). Under this account, older adults may struggle to translate their spatial knowledge into the survey-perspective output that the NARA requires. This interpretation aligns with the broader literature that aging specifically impairs the ability to switch to using allocentric reference frames (Harris et al., 2012) and reduces flexibility in switching between reference frames more broadly (Colombo et al., 2017; Harris and Wolbers, 2014). The present design cannot fully separate whether this deficit lies in building allocentric knowledge itself or in transforming information between reference frame types. Since prior work has also shown that aging-related deficits in map-based navigation are partially dissociable from cognitive map formation post-navigation (Iaria et al., 2009), future work could reverse the transformation direction – for example, providing participants with a pre-constructed map and testing first-person navigation from it – to isolate the reference frame transformation component and clarify which process is more affected by aging.

Importantly, NARA performance was positively associated with all three primary navigation measures in both age groups (Figures 4B–D), with better *NavCity* navigation performance correlating with higher NARA scores. Correlations within each age group demonstrates that cognitive map formation ability explains substantial variance in navigation performance beyond the variance accounted for by age alone. Similar correlation patterns and strengths in both younger and older adults suggests that the cognitive processes linking allocentric spatial knowledge to navigation efficiency remain fundamentally similar across age groups, even though the absolute level of cognitive map formation ability declines with age. Taken together, these findings indicate that the ability to form and recall allocentric representations is a key mechanistic factor underlying aging-related navigation impairments, with individual differences in allocentric spatial processing contributing substantially to the heterogeneity observed in older adult navigation performance.

Methodologically, future work using the NARA could complement the current scoring rubric with a continuous Euclidean distance metric between marked and correct building locations. While the current rubric distinguishes qualitatively meaningful error types (e.g., target-identity switches, mirrored or rotated configural errors), a Euclidean metric would provide a more sensitive measure of fine-grained spatial accuracy. Reporting both metrics in tandem may strengthen future characterizations of allocentric knowledge formation.

### 4.4 Heterogeneity in cognitive map formation among older adults

A central finding of this study is that NARA scores subdivided the older adult cohort into two subgroups with different navigation profiles. When older adults were split into NARA-defined cohorts, a higher-performing subgroup (OA_High_) demonstrated navigation performance either comparable to younger adults, or intermediate between younger adults and the lower-performing subgroup (OA_Low_), while OA_Low_ showed substantially lower performance (Figures 5, 6). This heterogeneity aligns with growing evidence that aging-related impairment in navigation ability is not uniform across individuals and reveals distinctions between spatial knowledge and the efficiency with which that knowledge can be executed.

Substantial heterogeneity within older adult populations in cognitive performance (Hultsch et al., 2002, 2011; Morse, 1993) and spatial ability (Nagel et al., 2009) has been well documented. While it is evident that advancing age is accompanied by decline in allocentric navigation (Colombo et al., 2017; Gazova et al., 2012; Laczó et al., 2018; Rodgers et al., 2012), mounting evidence suggests that access to allocentric representations may be preserved in some older adults, dependent on available cues or task demands (Bécu et al., 2020, 2023; Ekstrom and Hill, 2023; McAvan et al., 2021; Zhong et al., 2017). Research on spatial working memory demonstrates that performance heterogeneity is particularly pronounced within older adult samples, with some older adults achieving performance levels within the range of younger adults without signs of compensatory brain activation (Nagel et al., 2009). Similarly, a study examining strategy switching in navigation found that aging-related differences were only evident when comparing younger adults to poorer-performing older adults, while higher-performing older adults demonstrated spatial abilities that did not demonstrably differ from their younger counterparts (Zhong et al., 2017). Other research has identified a subset of older adults who demonstrate superior, sometimes even youth-like spatial memory and navigational abilities (Zhou et al., 2023), challenging the assumption that cognitive decline is inevitable with aging.

Notably, a recent review on spatial navigation and memory suggests that aging-related variance in navigation cannot be completely accounted for by allocentric deficits, but they may result from the inability to flexibly switch between spatial representations or different strategies based on available cues and task demands (Ekstrom and Hill, 2023). Therefore, this study directly extends previous literature by demonstrating that individual differences in the ability to transform first-person navigation experiences into third-person, topological survey knowledge—or “cognitive maps”—account for substantial variance in navigation performance among older adults.

This heterogeneity in allocentric knowledge recall has implications for understanding which navigation abilities are preserved, or not, in higher-performing older adults. The pattern of group differences across outcome measures reveals a dissociation between measures that index spatial accuracy (i.e., distance travelled) versus those that reflect processing efficiency (i.e., speed). When examining path efficiency through distance traveled, higher-performing older adults performed similarly to younger adults, indicating that aging-related differences in spatial knowledge may be attenuated in older adults with preserved allocentric processing abilities. Lower-performing older adults showed significantly longer distances traveled compared to both younger adults and higher-performing older adults, indicating fundamental deficits in route selection and cognitive map formation. By contrast, all three groups (YA, OA_High_, and OA_Low_) performed differently from each other on average for speed, indexing aging-related differences in processing speed that are well-established in the broader cognitive aging literature (Eckert et al., 2010; Meng et al., 2019).

Navigation time has a distinct position in this dissociation. On average, all three groups also performed differently from each other on navigation time. However, the block-specific analysis (Results, Section 3.4.1) shows that YA and OA_High_ converged on navigation time in later blocks (Figure 5F), paralleling the convergence seen on distance traveled (Figure 5E) rather than the persistent group separation seen on speed (Figure 5D). This pattern suggests that navigation time is more closely related to path efficiency than to processing speed in our paradigm: when younger adults and higher-performing older adults achieved comparable path efficiency in later blocks (Figure 5E), their navigation times also became indistinguishable, while their speeds remained distinct. Speed therefore appears to be the cleanest marker of persistent aging-related processing differences, as it does not show the convergence pattern observed for navigation time and distance.

This finding is notable because distance traveled was the only primary outcome measure where higher-performing older adults achieved performance statistically indistinguishable from younger adults at the averaged level, suggesting that path efficiency may be a particularly sensitive marker for identifying older adults who maintain spatial processing capabilities comparable to younger adults. While navigation time converged in the block-specific analysis, this convergence was not preserved at the averaged level effect. Together, this dissociation demonstrates that cognitive map formation ability accounts for substantial variance in older adult navigation performance, specifically on measures of path efficiency.

Performance trajectories across repeated *NavCity* exposure blocks revealed additional nuances in these group differences. For speed, groups showed divergent learning patterns: in the first exposure block, only younger adults differed significantly from low-performing older adults, but by the second and third blocks, all three groups were statistically distinct from one another. This progressive differentiation may reflect compounding effects of aging-related processing speed limitations that become more apparent with repeated environmental exposure. In contrast, navigation time and distance traveled showed convergent patterns. While all groups differed initially in the first exposure block, younger adults and high-performing older adults became statistically indistinguishable by the second and third exposure. This convergence suggests that high-performing older adults can achieve comparable spatial efficiency to younger adults with repeated exposure, particularly when performance is indexed by path optimization rather than speed of execution. This analysis further supports the notion that distance traveled, as a measure of spatial accuracy, distinguishes between older adults who maintain versus lose fundamental wayfinding ability – abilities that depend on the formation and use of cognitive maps and that may signal broader trajectories of aging-related cognitive change.

Importantly, these differences between higher- and lower-performing older adults could not be accounted for by differences in age or any other measured demographic, cognitive, or health-related factor. This pattern suggests that the NARA-based subdivision may specifically reflect allocentric spatial processing abilities rather than demographic characteristics or broader differences in general cognitive function.

A complementary analysis within OA subgroups showed that NARA was not significantly associated with any primary *NavCity* outcome within either OA_High_ or OA_Low_ subgroup (Section 3.4.3, Supplementary Figure S1). Together, along with the full cohort correlations (Figures 4B–D), this pattern indicates that the NARA–*NavCity* relationship is best characterized as a continuous association across the full OA range, rather than a reflection of distinct within-subgroup gradients.

Additionally, post-hoc correlation analyses showed that measures which were significantly different between younger and older adults—including outcomes representing processing speed (Trails A, Trails B), cognitive set-shifting (Trails B-A), visuospatial working memory (Corsi Blocks), self-reported navigational confidence (SBSOD), and technology experience (VR experience, video game usage)—were not associated with any of the primary *NavCity* navigation measures (speed, distance traveled, or navigation time) averaged across blocks (all p_corr_ > 0.05). This suggests that aging-related navigation deficits observed in *NavCity* were not primarily attributable to declining general cognitive abilities (i.e., processing speed, set-shifting cost), reduced technology familiarity (i.e., VR experience, video game usage), nor lower self-reported navigational confidence, but rather reflect deficits more specific to spatial navigation processes.

Taken together, these findings demonstrate that individual differences in the transformation of spatial information into allocentric knowledge identified a “preserved” profile of navigation ability in a subgroup of older adults – achieveing path efficiency comparable to younger adults despite some persistent aging-related slowing in processing speed – independent of chronological age within our older adult sample. This dissociation highlights the importance of distinguishing between path efficiency (indexed primarily by distance traveled) and processing speed (indexed by speed) when assessing aging-related changes and underscores the important role of individual differences in allocentric knowledge in understanding navigation heterogeneity among older adults.

## 5 Conclusion

This study investigated aging-related navigation performance in immersive virtual reality, across multiple within-session exposures. Our findings reveal three key insights: older adults showed lower performance than younger adults across multiple navigation measures; both age groups demonstrated similar rates of improvement with repeated exposure; and importantly, substantial heterogeneity within older adults emerged based on the ability to form and recall allocentric spatial representations. High-performing older adults achieved spatial accuracy comparable to younger adults despite aging-related slowing in efficiency metrics, while low-performing older adults showed widespread deficits. These findings demonstrate that individual differences in cognitive mapping ability may predict navigation performance more effectively than chronological age alone, suggesting that preserved allocentric spatial processing may protect against navigation decline in some older adults.

These findings have important clinical implications. Current assessments of aging-related navigational decline often treat older adults homogeneously, potentially masking meaningful individual differences that could inform interventions. By identifying older adults with impaired allocentric knowledge formation, clinicians could target individuals most at risk for real-world navigation difficulties and spatial disorientation, such as early warning signs of mild cognitive impairment or Alzheimer’s disease. Furthermore, understanding that some older adults maintain robust cognitive mapping abilities despite general aging-related slowing suggests that spatial training, environmental enrichment, or cognitive rehabilitation may benefit those who struggle with map formation.

Several directions for future research emerge from these findings. First, extending the number of exposures would clarify whether performance between high-performing older adults and younger adults would fully converge with sufficient practice, or whether persistent differences in speed reflect fundamental constraints of cognitive aging. Extended exposure paradigms could also reveal whether lower-performing older adults show delayed but eventual improvement in allocentric knowledge formation, suggesting intact learning mechanisms that simply require more exposure. Second, neuroimaging studies are needed to identify the neural correlates that distinguish higher- from lower-performing older adults. Understanding the neural underpinnings of observed differences would clarify whether behavioral measures like NARA serve as reliable markers of underlying brain health and aging-related neurodegeneration. Third, longitudinal studies should examine whether allocentric knowledge phenotypes remain stable over time or predict trajectories of cognitive decline. Given the cross-sectional nature of the study, we were unable to determine specific, longitudinal aging-effects within a given individual. However, our current results support future studies to characterize differences in long-term aging-related trajectories of spatial navigation ability, which may potentially serve as early behavioral biomarkers for at-risk individuals. Finally, intervention studies can test whether targeted spatial cognitive training can improve allocentric knowledge formation in lower-performing older adults, thereby enhancing navigation efficiency and potentially supporting broader spatial cognitive health. Together, these research directions would advance both theoretical understanding of cognitive aging heterogeneity and practical approaches to maintaining spatial independence in older adulthood.

## Data Availability

The datasets presented in this study can be found in online repositories. The names of the repository/repositories and accession number(s) can be found at: https://osf.io/qmwyk.
