# Supplementary material for: Distinct aging-related profiles of allocentric knowledge recall following navigation in an immersive, naturalistic, city-like environment
Source: Front Aging Neurosci. 2026 Jun 2;18:1746016. doi: 10.3389/fnagi.2026.1746016 (PMC13269101; doi:10.3389/fnagi.2026.1746016)
Supplement: Supplementary file 1 [file Table_1.docx]

**Supplementary Table 1**

*Participant Characteristics & Cognitive Performance.*

Data are shown for younger adults (YAs, n = 30) and older adults (OAs, n = 30), with means **±** sample SDs at the bottom of each section. Older adult participants are further grouped into NARA-defined subgroups (OA_High_, n = 16; OA_Low_, n = 14), separated by a horizontal divider, with subgroup means **±** standard deviations for each group. Trails B and the set-shifting composite (Trails B−A) are reported in the Results.

| **Subject ID** | | **Age** (yrs) | **Gender** (W, M, NB) | **Handedness** (R, L, M) | **VR Experience**  (0-2 score) | **Game Usage**  (hrs / wk) | **Exercise Frequency** (hrs / wk) | **PSQI**  (total score) | **SSQ**  (Post - Pre) | **MiniCog** (0-5 score) | **SBSOD**  (1-7 score) | **Trails Making A** (sec**)** | **Corsi Blocks**  (total) |
| --- | --- | --- | --- | --- | --- | --- | --- | --- | --- | --- | --- | --- | --- |
| *YA* | *S01* | *24.7* | *W* | *R* | *0* | *0* | *6* | *5* | *5* | *5* | *2.20* | *30.3* | *74* |
|  | *S02* | *26.3* | *W* | *R* | *2* | *10* | *10* | *5* | *0* | *5* | *3.40* | *20.9* | *96* |
|  | *S03* | *19.7* | *M* | *M* | *2* | *2* | *2* | *3* | *0* | *5* | *5.20* | *26.8* | *92* |
|  | *S04* | *22.7* | *W* | *R* | *1* | *0* | *5* | *6* | *3* | *4* | *5.27* | *32.3* | *93* |
|  | *S05* | *28.7* | *W* | *R* | *1* | *0* | *3* | *6* | *6* | *5* | *4.87* | *30.2* | *85* |
|  | *S06* | *27.3* | *M* | *R* | *1* | *7* | *5* | *4* | *3* | *5* | *4.53* | *24.1* | *84* |
|  | *S07* | *30.0* | *M* | *R* | *0* | *5* | *4.5* | *8* | *-2* | *4* | *3.53* | *30.5* | *100* |
|  | *S08* | *25.3* | *W* | *L* | *1* | *0* | *3* | *3* | *1* | *5* | *3.93* | *24* | *88* |
|  | *S09* | *25.6* | *W* | *M* | *0* | *0* | *2.5* | *3* | *-2* | *5* | *5.40* | *23.7* | *79* |
|  | *S10* | *28.8* | *M* | *R* | *2* | *3* | *3* | *7* | *-1* | *5* | *5.60* | *23.6* | *110* |
|  | *S11* | *23.6* | *M* | *R* | *2* | *3* | *3* | *5* | *0* | *5* | *3.60* | *21* | *85* |
|  | *S12* | *21.4* | *W* | *R* | *0* | *3* | *0* | *5* | *2* | *5* | *2.67* | *25.7* | *105* |
|  | *S13* | *26.2* | *W* | *R* | *1* | *1* | *4.5* | *5* | *0* | *5* | *4.47* | *22.7* | *115* |
|  | *S14* | *24.5* | *M* | *R* | *2* | *10* | *15* | *6* | *1* | *4* | *3.40* | *17.1* | *87* |
|  | *S15* | *21.4* | *W* | *M* | *0* | *0* | *1.2* | *5* | *8* | *5* | *3.00* | *26.1* | *81* |
|  | *S16* | *26.5* | *W* | *R* | *0* | *0* | *11* | *2* | *-3* | *5* | *3.87* | *21.3* | *88* |
|  | *S17* | *25.9* | *W* | *R* | *0* | *0* | *3.5* | *3* | *0* | *5* | *5.33* | *27* | *99* |
|  | *S18* | *21.6* | *W* | *R* | *0* | *7.5* | *3* | *5* | *-1* | *5* | *4.27* | *25* | *88* |
|  | *S19* | *24.3* | *M* | *R* | *2* | *0* | *14* | *4* | *4* | *5* | *6.00* | *29.5* | *84* |
|  | *S20* | *26.0* | *W* | *R* | *0* | *0* | *3.5* | *4* | *2* | *5* | *5.40* | *35.2* | *84* |
|  | *S21* | *26.6* | *W* | *R* | *2* | *3* | *0.5* | *6* | *0* | *5* | *2.13* | *32.3* | *95* |
|  | *S22* | *27.8* | *M* | *R* | *0* | *4* | *4* | *5* | *-3* | *5* | *5.47* | *22.9* | *66* |
|  | *S23* | *22.3* | *W* | *R* | *2* | *0* | *2* | *12* | *3* | *5* | *4.87* | *25* | *79* |
|  | *S24* | *20.9* | *W* | *R* | *1* | *0* | *6* | *6* | *0* | *5* | *3.00* | *23.9* | *95* |
|  | *S25* | *20.3* | *W* | *L* | *0* | *0* | *5* | *8* | *-1* | *5* | *5.60* | *22.9* | *82* |
|  | *S26* | *18.8* | *M* | *R* | *2* | *2.5* | *7.5* | *4* | *0* | *5* | *4.00* | *33.8* | *94* |
|  | *S27* | *24.5* | *M* | *R* | *2* | *0* | *5* | *3* | *3* | *4* | *5.00* | *20.6* | *83* |
|  | *S28* | *21.3* | *M* | *R* | *2* | *7* | *10* | *3* | *0* | *5* | *5.93* | *22.7* | *99* |
|  | *S29* | *22.8* | *M* | *R* | *0* | *3* | *6* | *4* | *0* | *5* | *4.67* | *23.2* | *84* |
|  | *S30* | *24.3* | *M* | *R* | *2* | *10* | *6* | *6* | *2* | *4* | *3.93* | *33.2* | *82* |
| ***YA: Mean ± SD*** | | ***24.3 ± 2.88*** | ***W:17*** | ***R:25*** | ***0.93 ±***  ***0.87*** | ***2.70 ± 3.39*** | ***4.69 ±***  ***3.30*** | ***5.1 ± 1.95*** | ***1.00 ±***  ***3.31*** | ***4.80 ± 0.48*** | ***4.35 ± 1.10*** | ***25.92 ± 4.56*** | ***89.2 ± 10.43*** |
| *OA_High_* | *S31* | *61.3* | *M* | *R* | *0* | *0* | *1.5* | *6* | *3* | *5* | *6.87* | *35.7* | *78* |
|  | *S33* | *69.1* | *W* | *R* | *1* | *0* | *4.5* | *7* | *2* | *4* | *4.00* | *23.6* | *65* |
|  | *S34* | *65.6* | *W* | *R* | *1* | *0* | *4* | *4* | *-2* | *5* | *4.53* | *28.2* | *75* |
|  | *S35* | *60* | *W* | *R* | *0* | *0* | *2* | *6* | *-3* | *4* | *5.00* | *29.6* | *77* |
|  | *S38* | *73.8* | *M* | *M* | *0* | *0* | *7* | *3* | *0* | *3* | *5.93* | *45.0* | *66* |
|  | *S39* | *65.6* | *W* | *R* | *2* | *0* | *5* | *9* | *0* | *4* | *5.87* | *36.2* | *82* |
|  | *S40* | *65.4* | *W* | *L* | *0* | *0* | *3* | *1* | *0* | *5* | *5.07* | *25.9* | *76* |
|  | *S41* | *64.6* | *W* | *R* | *0* | *0* | *2* | *1* | *0* | *4* | *5.07* | *36.7* | *54* |
|  | *S43* | *71.1* | *M* | *R* | *0* | *0* | *2.5* | *6* | *4* | *4* | *6.87* | *35.9* | *75* |
|  | *S45* | *69.9* | *W* | *R* | *0* | *2* | *2* | *2* | *-3* | *4* | *3.60* | *36.2* | *64* |
|  | *S46* | *61.7* | *M* | *R* | *1* | *0* | *0* | *8* | *9* | *5* | *5.53* | *22.4* | *87* |
|  | *S49* | *74.4* | *W* | *R* | *0* | *0* | *5* | *9* | *4* | *4* | *5.53* | *36.5* | *61* |
|  | *S53* | *72.4* | *W* | *R* | *0* | *0* | *10.5* | *5* | *0* | *5* | *4.93* | *40.7* | *80* |
|  | *S54* | *74.2* | *M* | *R* | *0* | *0* | *9* | *8* | *0* | *5* | *5.27* | *34.9* | *73* |
|  | *S55* | *72.0* | *W* | *R* | *1* | *0* | *14* | *4* | *0* | *5* | *5.00* | *34.7* | *58* |
|  | *S57* | *63.5* | *M* | *R* | *0* | *0* | *10* | *0* | *0* | *5* | *5.40* | *34.1* | *75* |
| ***OA_High_: Mean ± SD*** | | ***67.79 ± 4.90*** | ***W:10*** | ***R:14*** | ***0.38 ±***  ***0.62*** | ***0.13 ± 0.50*** | ***5.13 ±***  ***3.93*** | ***4.94 ± 2.93*** | ***0.88 ± 3.01*** | ***4.5 ± 0.63*** | ***5.28 ± 0.87*** | ***33.52 ± 6.08*** | ***71.63 ± 9.24*** |
| OA_Low_ | *S32* | *78.8* | *M* | *M* | *1* | *0* | *7* | *3* | *-1* | *3* | *6.13* | *47.6* | *54* |
|  | *S36* | *65.8* | *W* | *R* | *0* | *8* | *0* | *7* | *8* | *4* | *3.53* | *29.5* | *66* |
|  | *S37* | *74.3* | *W* | *R* | *0* | *0* | *6* | *8* | *0* | *5* | *5.27* | *36.8* | *60* |
|  | *S42* | *70.3* | *M* | *L* | *0* | *0* | *3* | *4* | *0* | *4* | *5.27* | *27.6* | *76* |
|  | *S44* | *82.2* | *W* | *R* | *0* | *0* | *3.5* | *11* | *2* | *3* | *5.20* | *48.6* | *63* |
|  | *S47* | *65.4* | *W* | *R* | *1* | *0* | *3* | *1* | *-2* | *5* | *5.13* | *34.6* | *67* |
|  | *S48* | *76.2* | *W* | *R* | *0* | *0* | *8* | *2* | *1* | *5* | *5.53* | *32.4* | *75* |
|  | *S50* | *70.8* | *M* | *R* | *0* | *0* | *5.25* | *2* | *-1* | *3* | *4.27* | *31.5* | *71* |
|  | *S51* | *67.2* | *W* | *R* | *1* | *0* | *5* | *7* | *7* | *4* | *4.27* | *31.4* | *73* |
|  | *S52* | *69.3* | *M* | *R* | *0* | *0* | *5* | *7* | *-2* | *3* | *3.87* | *47.9* | *60* |
|  | *S56* | *61.2* | *W* | *R* | *1* | *0* | *7* | *4* | *1* | *5* | *3.27* | *24.3* | *85* |
|  | *S58* | *72.0* | *M* | *R* | *1* | *0* | *3* | *2* | *1* | *5* | *4.13* | *35.2* | *83* |
|  | *S59* | *72.3* | *W* | *R* | *1* | *0* | *8* | *6* | *0* | *5* | *6.07* | *34.4* | *58* |
|  | *S60* | *60.0* | *W* | *R* | *1* | *7* | *4.5* | *2* | *3* | *5* | *6.13* | *28.6* | *89* |
| ***OA_Low_: Mean ± SD*** | | ***70.41 ± 6.30*** | ***W:9*** | ***R:12*** | ***0.50 ±***  ***0.52*** | ***1.07 ± 2.73*** | ***4.88 ±***  ***2.26*** | ***4.71 ± 2.97*** | ***1.21 ± 3.02*** | ***4.36 ± 0.84*** | ***4.86 ± 0.97*** | ***35.03 ± 7.77*** | ***70.00 ± 10.74*** |
| ***OA: Mean ± SD*** | | ***69.03 ± 5.66*** | ***W:18*** | ***R:26*** | ***0.33 ± 0.55*** | ***0.57 ± 1.92*** | ***5.04 ± 3.19*** | ***4.83 ± 2.90*** | ***0.93 ± 4.35*** | ***4.43 ± 0.73*** | ***5.08 ± 0.93*** | ***34.22 ± 6.84*** | ***70.87 ± 9.83*** |
