# Supplementary figures and images for: Distinct aging-related profiles of allocentric knowledge recall following navigation in an immersive, naturalistic, city-like environment
Source: Front Aging Neurosci. 2026 Jun 2;18:1746016. doi: 10.3389/fnagi.2026.1746016 (PMC13269101; doi:10.3389/fnagi.2026.1746016)

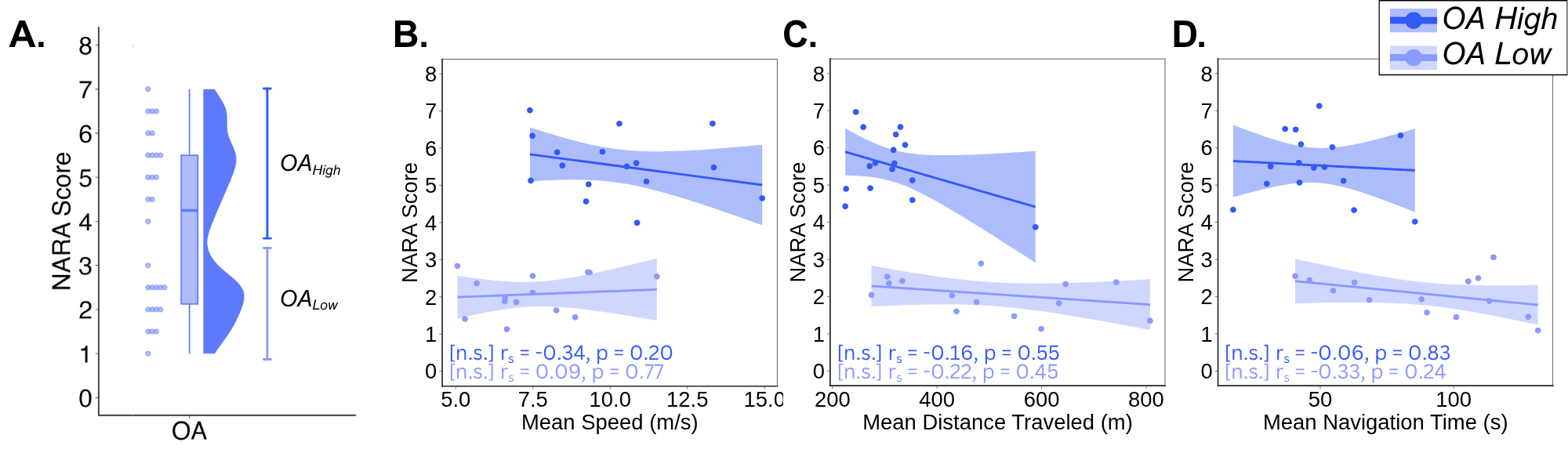

Supplement: Supplementary file 2 [file Image_1.PNG]
